# Intertidal Warfare: Synergistic Allelopathy Mediates Spatial Competition between Two Marine Calcareous‐Shelled Sessile Organisms

**DOI:** 10.1002/advs.202512644

**Published:** 2025-12-07

**Authors:** Zhuo Li, Zixin Huo, Shanshan Yao, Xianmeng Liang, Yiran Zhao, Yanxin Wang, Shifeng Guo, Caihuan Ke, Pei Su, Danqing Feng

**Affiliations:** ^1^ State Key Laboratory of Mariculture Breeding College of Ocean and Earth Sciences, Xiamen University Xiamen Fujian 361102 China; ^2^ Shenzhen Key Laboratory of Smart Sensing and Intelligent Systems Shenzhen Institute of Advanced Technology, Chinese Academy of Sciences Shenzhen Guangdong 518055 China; ^3^ Laoshan laboratory Qingdao 266237 China

**Keywords:** allelopathy, attachment, chemical ecology, marine sessile organisms, synergistic effect

## Abstract

Chemical warfare among marine sessile organisms remains poorly understood. Chemical defense in calcareous‐shelled organisms in particular has been largely neglected, yet this may be important in spatial dominance of crowded intertidal ecosystems. Using field survey data, spatial competition in intertidal zones between two calcareous‐shelled sessile species are discovered, the barnacle *Balanus albicostatus* and the mussel *Vignadula atrata*. Using chemical analysis and bioassays, it is found that *B. albicostatus* releases chemical cues with inhibitory activity against the attachment of *V. atrata*. This allelochemical is identified as a blend of palmitic acid (PA) and 1‐palmitoyl‐sn‐glycero‐3‐phosphocholine (PGPC) in a synergistic and unique ratio (1:1.92). This mixture of PA and PGPC synergistically reduced byssus thread production, adhesive plaque area and adhesion force of mussel foot proteins (MFPs) in *V. atrata*. Further analysis showed that this mixture down‐regulated expression of the genes associated with byssus formation and adhesion (*PreCol‐NG*, *MFP2*, *MFP11, Tyr*, *BPP4*, and *PPO*) and led to a lower activity of the enzyme polyphenol oxidase essential to mussel attachment, implying an underlying mechanism by which allelochemicals inhibit mussel attachment. This underlines the importance of allelopathy in interspecies competition between calcareous‐shelled sessile organisms and provides information which may be useful for developing novel biofouling control systems.

## Graphical abstract

This study reveals a hidden chemical weapon among calcareous‐shelled sessile organisms in the intertidal zone: the barnacle *Balanus albicostatus* releases a synergistic blend of palmitic acid and 1‐palmitoyl‐sn‐glycero‐3‐phosphocholine as the allelochemical with the inhibitory activity against attachment of the mussel *Vignadula atrata*, which may mediate the spatial competition between these two marine species.

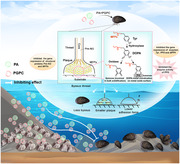

## 1 Introduction

Chemical communication is centrally important in biology.^[^ ^1^ ^]^ Chemical signals play pivotal roles in regulating intra‐ and interspecies interactions such as conspecific aggregation, mate recognition, food location, and prey‐predator interactions. These processes in turn critically affect species demography, community structure, and ecosystem function in both terrestrial and marine ecosystems.^[^ ^2^, ^3^, ^4^, ^5^ ^]^ Compared to many well‐described infochemicals found in terrestrial ecosystems, the identity, ecological importance, and action mechanism of marine infochemicals remain poorly understood.^[^ ^6^ ^]^ In the marine environment, many of the more primitive organisms, without advanced visual or auditory abilities, rely largely on chemicals to regulate biotic interactions.^[^ ^7^, ^8^ ^]^ Therefore, improving our understanding of how these cues function is crucial for a better understanding of marine ecosystems.

Like terrestrial plants, marine sessile organisms lack the ability to change their location when faced with predators or competitors.^[^ ^9^ ^]^ For those sessile, soft‐bodied organisms without calcareous shells, such as sponges, soft corals and seaweeds, chemical defense plays an important role in their survival strategy.^[^ ^10^ ^]^ This topic has received much attention, although only a few studies have characterized metabolites with an ecological role in defense.^[^ ^11^, ^12^, ^13^ ^]^ On the other hand, relatively little is known about the chemical defense of marine sessile organisms with calcareous shells such as mussels and barnacles. Organisms with protective calcareous shells are often assumed to rely on physical rather than chemical defense. This raises the question of whether chemical defense occurs at all in sessile organisms with calcareous shells? If it does, how might these chemical interactions shape the ecology of these organisms?

As a fundamental requirement for life, space in which to live is often a limiting resource in marine hard substratum communities.^[^ ^14^ ^]^ Especially for marine sessile invertebrates, space is needed for settlement and colonization. Competition for space is therefore particularly high on hard substrata, such as coral reefs and rocky intertidal shores.^[^ ^15^ ^]^ The production and release of compounds to inhibit competitor species, known as allelopathy,^[^ ^16^ ^]^ is important for spatial competition in coral reef ecosystems and has been described for sponge‐coral interactions,^[^ ^17^ ^]^ seaweed‐coral interactions,^[^ ^18^ ^]^ and interactions between different coral species.^[^ ^9^ ^]^ However, allelopathy in rocky intertidal ecosystems, particularly among benthic organisms with calcareous shells, has not previously been observed. Barnacles and mussels are common inhabitants of rocky intertidal shores. Their strong adherence to surfaces not only enables them to endure tidal and wave action, but also makes them major fouling organisms causing huge economic losses worldwide.^[^ ^19^, ^20^ ^]^

Many barnacles, especially the free‐living barnacles, have a planktonic larval phase which typically includes nauplius and cyprid stages. The cyprid stage is the critical stage for searching a suitable substratum upon which to settle. Cyprids settle permanently and metamorphose into sessile juveniles. For mussels, they attach themselves to surfaces using a proteinaceous structure, called the byssus. This is composed of a thread and an adhesive plaque. The thread connects the soft mussel tissues to surfaces via the adhesive plaque at its distal end, and the plaque is responsible for mussel adhesion.^[^ ^21^ ^]^ Mussels foot proteins (MFPs, see Table S1, Supporting Information of Abbreviations) are found in the plaque and in the cuticle surrounding the byssus.^[^ ^22^ ^]^ As well as strongly attaching to rocks, adult mussels can also release their byssus threads and free themselves from substrates to change positions, thus demonstrating limited mobility.^[^ ^23^ ^]^ Despite this, for mussels living in dense aggregations, cross adhesion by the byssal threads of other adults often renders individuals immobile. Nevertheless, the ability of byssus release over time can enable mussels to gradually relocate away from unfavorable environments.^[^ ^23^, ^24^, ^25^, ^26^, ^27^, ^28^ ^]^ On the other hand, adult barnacles which attach to rock surfaces as adults, lack the ability to move once attached. Mussels and barnacles usually coexist in rocky intertidal habitats, and we therefore hypothesized that allelochemicals may be involved in their spatial competition. For barnacles which are unable to move physically, allelochemical production may have provided a greater selective advantage.

To explore this hypothesis, we first sought to understand if spatial competition could be observed by undertaking a preliminary field study on the southeast coast of China, where it was observed that areas with more barnacles *Balanus* (= *Fistulobalanus*) *albicostatus* had lower populations of the mussel *Vignadula atrata*, and conversely, the regions with less *B. albicostatus* had more *V. atrata*. These two species are both filter feeders and are of a similar size (0.6–1.4 cm), and are widely distributed along the rocky intertidal zones of East Asia. Here, we present detailed and quantitative field data that indicate a competitive relationship between these two species. Furthermore, we report the identification of the specific allelochemicals mediating this interaction, and present molecular evidence for the mode of action of these molecules. Our discovery not only reshapes our understanding of competition among marine calcareous‐shelled sessile organisms but also underscores the intricate mechanisms governing community structure in rocky intertidal ecosystems. The present work opens up new avenues for exploring control strategies to prevent marine biofouling, a global challenge costing billions annually,^[^ ^20^ ^]^ and also offers a framework for studying similar interactions among other species.

## 2 Results

### 2.1 Results of the Field Survey

**Figure 1:**
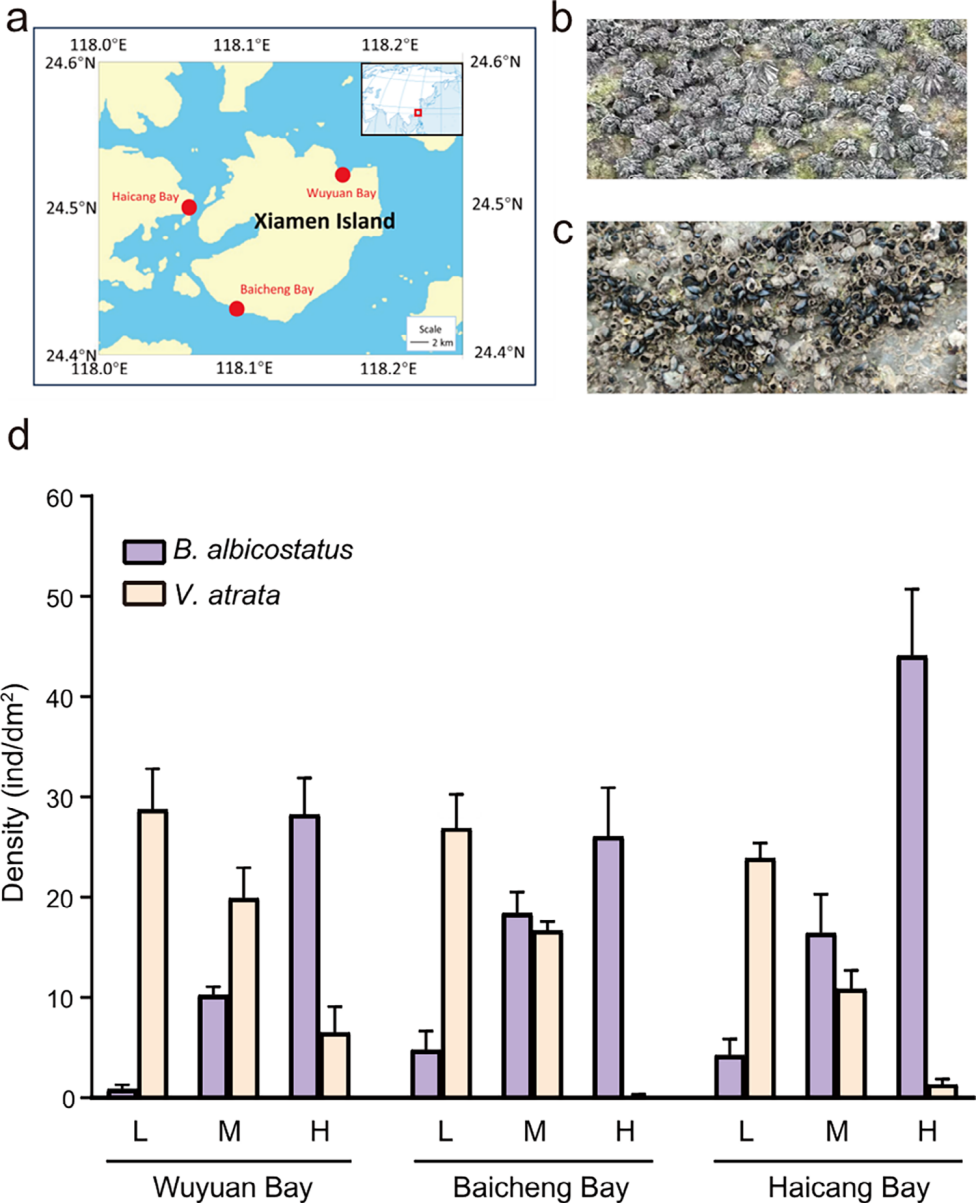
Results of the field survey. a) Survey locations for *B. albicostatus* and *V. atrata* (Wuyuan Bay, Baicheng Bay, and Haicang Bay, indicated by red dots). b) Survey locations with a high density of *B. albicostatus*. c) Survey locations with high density of *V. atrata*. d) The inverse relationship between the density of *B. albicostatus* and that of *V. atrata* in the three locations. L: In zones with low density of *B. albicostatus*; M: In zones with medium density of *B. albicostatus*; H: In zones with high density of *B. albicostatus*. Data shown are means ± SE of replicates (*n* = 3).

Field observations revealed that there is an inverse relationship between the density of *V. atrata* and that of *B. albicostatus*. In Baicheng Bay, when the density of *B. albicostatus* increased from 4.75 to 18.42 ind cm^−2^, *V. atrata* decreased from 26.92 to 16.67 ind cm^−2^ (*P* < 0.05, **Figure** **1**d). When the density of *B. albicostatus* continued to increase from 18.42 to 26.08 ind cm^−2^, the density of *V. atrata* continued to decrease from 16.67 to 0.17 ind cm^−2^ (Figure 1d). The observation that an increase in *B. albicostatus*, corresponds to a decrease in *V. atrata* was also found in the other two bays (Figure 1d), suggesting that there is competitive exclusion between these species. Correlation analysis showed that the correlation coefficients between the densities of *B. albicostatus* and *V. atrata* were mostly close to ‐1 in all three locations (Table S2, Supporting Information), confirming the highly negative correlation between these two species.

### 2.2 *B. albicostatus* Release Chemical Cues to Inhibit Attachment of *V. atrata*

**Figure 2:**
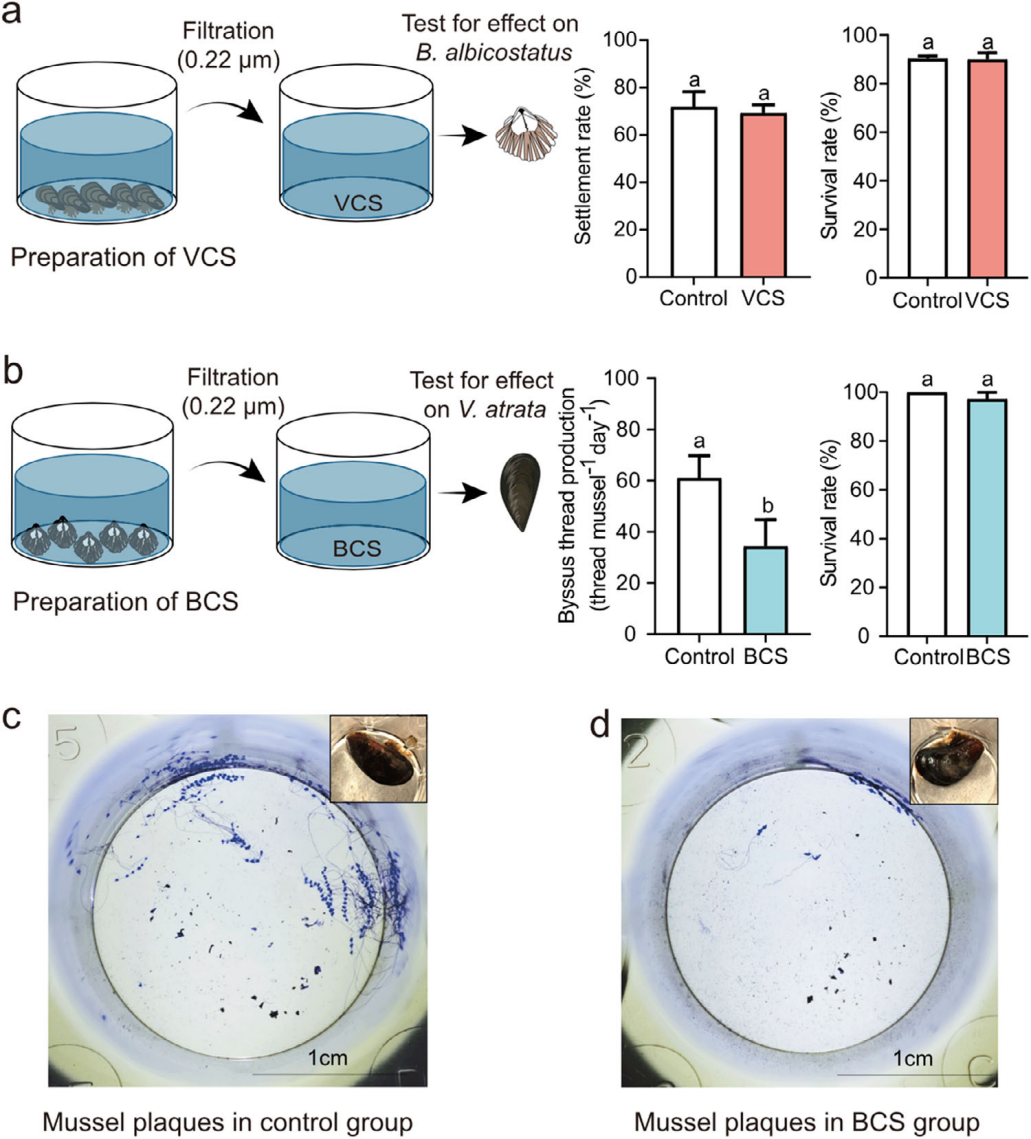
*B. albicostatus* adults release chemical cues that inhibit *V. atrata* attachment. a) Preparation of VCS and its effect on settlement and survival of *B. albicostatus* cyrpids after 24‐h exposure. b) Preparation of BCS and its effect of on byssus thread production and survival of *V. atrata* after 24‐h exposure. c) The byssus plaques (stained with Coomassie brilliant blue) of *V. atrata* cultured under control conditions. d) The byssus plaques of *V. atrata* cultured in the presence of BCS. Photos of corresponding *V. atrata* individuals which produced these byssus plaques are shown in the upper right corner of (c,d). The preparation of BCS and VCS is described in the Methods. Control: filtered (0.22 µm) seawater. Data shown are means ± SE of replicates (*n* = 3). Different lower‐case letters above bars represent significant differences (Student's t‐test, *P* < 0.05).

To examine whether the competitive relationship between *V. atrata* and *B. albicostatus* may be mediated by secreted chemical cues, we tested the effects of their adult‐conditioned seawater on the settlement and mortality of each other. As shown in **Figure** **2** and *V. atrata* conditioned seawater (VCS) showed no significant impact (*P* = 0.5637) on the settlement of *B. albicostatus* cyprids (mean = 69.1867 ± 6.08%, *n* = 3) compared with the control (mean = 71.95 ± 11.03%, *n* = 3), while *B. albicostatus* conditioned seawater, as indicated by BCS, significantly inhibited the byssus thread production of *V. atrata* (*P* < 0.05), without any toxic effect on its survival rate. These findings indicated that *B. albicostatus* is able to release chemical cues into seawater with inhibitory activity against *V. atrata* attachment.

### 2.3 Palmitic Acid (PA) and 1‐Palmitoyl‐sn‐glycero‐3‐phosphocholine (PGPC) Released by *B. albicostatus* Synergistically Inhibit Attachment of *V. atrata*

**Figure 3:**
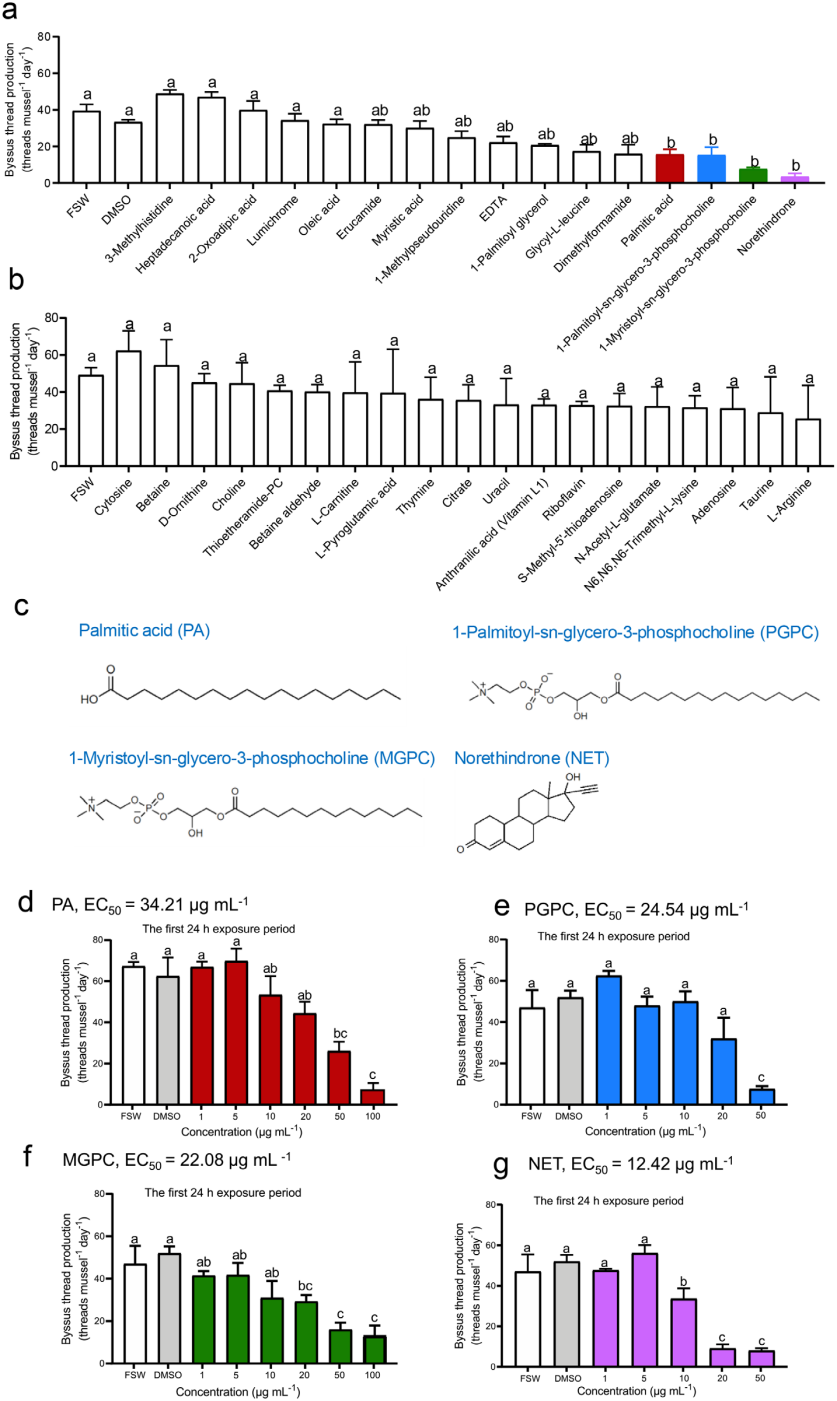
Inhibitory compounds against *V. atrata* attachment in the MCF of *B. albicostatus*. a) Effect of 16 lipophilic compounds (among the top 35 compounds in *B. albicostatus* MCF) on byssus thread production of *V. atrata*. b) Effect of 19 hydrophilic compounds (among the top 35 compounds in *B. albicostatus* MCF) on byssus thread production of *V. atrata*. c) Chemical structures of the four inhibitory compounds. d–g) Byssus thread production of *V. atrata* following exposure to PA, MGPC, PGPC, and NET for a 24 h period (exposure effect). FSW: The filtered seawater control. DMSO: The solvent only control (0.5% dimethyl sulfoxide in FSW). Data shown are means ± SE of replicates (*n* = 3). Different lower‐case letters above bars represent significant differences (Dunnett's test, *P* < 0.05). In Figure 3a,d–g, the DMSO group was used as the control for comparison of treatments using Dunnett's test. In Figure 3b, the FSW group was used as the control for comparison of treatments using Dunnett's test.

Since barnacles release substances from their mantle cavity fluid (MCF), we conducted chemical analysis of the MCF of *B. albicostatus* using Liquid Chromatography–Tandem Mass Spectrometry (LC‐MS/MS), which revealed the presence of 181 different compounds in varying abundance (Figure S2 and Table S3, Supporting Information). From these 181 compounds, we selected compounds that could be purchased commercially and ranked them by relative abundance in MCF from high to low. The top 35 compounds (Table S4, Supporting Information) were then tested individually at a concentration of 50 µg mL^−1^ for their effect on byssus thread production of *V. atrata*. As shown in **Figure** **3**a,b, of the 35 compounds tested, there were four which significantly inhibited byssus thread production (*P* < 0.05): PA, norethindrone (NET), 1‐myristoyl‐sn‐glycero‐3‐phosphocholine (MGPC), and PGPC. Their chemical structures are shown in Figure 3c. Furthermore, a series of concentrations (1–50 µg mL^−1^) of these four compounds were tested for inhibitory activity and their EC_50_ values were calculated, which ranged from 12.42 to 34.21 µg mL^−1^ (Figure 3d–g). It was noteworthy that exposure to these four compounds had no obvious residual effects on attachment of *V. atrata*. When these four compounds were removed, byssus thread production in each treatment was not significantly different from the control (Figure S3, Supporting Information), suggesting that their inhibitory effect on mussel attachment is reversible.

**Figure 4:**
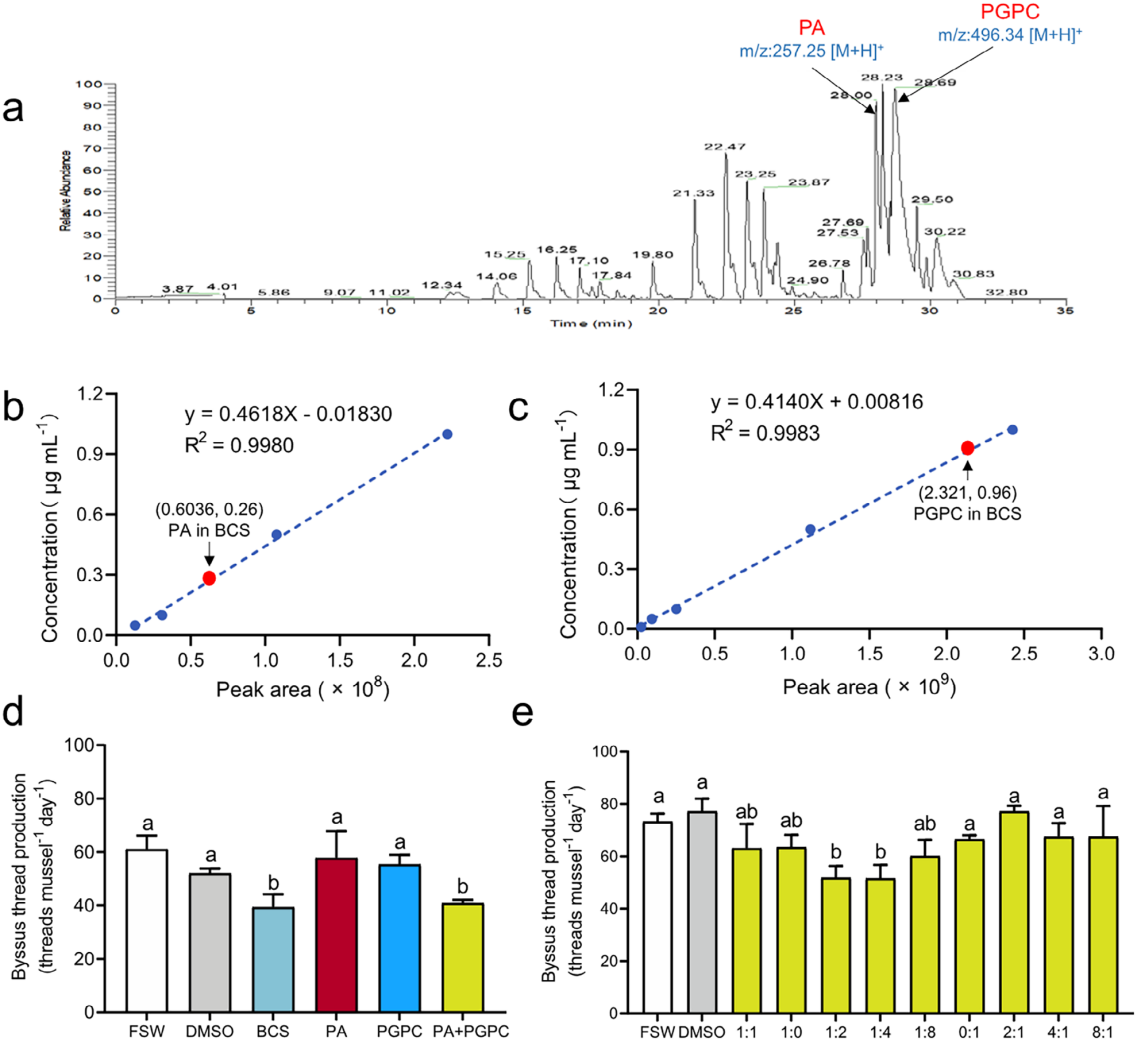
PA and PGPC released from *B. albicostatus* synergistically inhibited attachment of *V. atrata*. a) Detection by liquid chromatography‐mass spectrometry of PA and PGPC in BCS. b) Standard curve of PA and determination of PA concentration in BCS (0.26 µg mL^−1^, the red dot on the curve). c) Standard curve of PGPC and determination of PA concentration in BCS (0.96 µg mL^−1^, the red dot on the curve). d) Byssus thread production of *V. atrata* in response to 0.26 µg mL^−1^ PA, 0.96 µg mL^−1^ PGPC and a mixture of the two. e) Byssus thread production of *V. atrata* in response to different ratios of PA and PGPC. Here the concentration (µm) ratio was PA: PGPC. The different ratio treatments were at the same total concentration of compounds (3 µm). FSW: The FSW control. DMSO: The solvent only control (0.5% DMSO in FSW). BCS was used as the positive control. Data shown are means ± SE of replicates (*n* = 3). Different lower‐case letters above bars represent significant differences (Dunnett's test, *P* < 0.05). The DMSO group was used as the control for comparison of treatments using Dunnett's test.

To serve as effective cues in the natural environment, these four active compounds should be released into seawater by *B. albicostatus* at concentrations that are effective at inhibiting *V. atrata* attachment. Therefore, we measured their concentrations in *B. albicostatus* conditioned seawater by Liquid Chromatography Mass Spectrometry (LC‐MS), and found that PA and PGPC are present in BCS at concentrations of 0.26 and 0.96 µg mL^−1^, respectively (**Figure** **4**a–c; Figure S4, Supporting Information), while MGPC and NET were not detected in BCS. As shown in Figure 4d, PA at 0.26 µg mL^−1^ showed no inhibitory activity against *V. atrata* attachment, neither did PGPC at 0.96 µg mL^−1^ (which was consistent with the findings shown in Figure 3d,e). However, a mixture of 0.26 µg mL^−1^ PA (1.01 µm) and 0.96 µg mL^−1^ PGPC (1.94 µm) significantly inhibited the byssus thread production of *V. atrata* (*P* < 0.05), indicating a synergistic effect of these two compounds. Furthermore, it was found that this mixture was equally effective as BCS at inhibiting byssus thread production (Figure 4d), suggesting that PA and PGPC appeared to be responsible for the inhibitory activity of BCS. This mixture (0.26 µg mL^−1^ PA + 0.96 µg mL^−1^ PGPC) will hereafter be abbreviated as “PA+PGPC”.

To determine whether there is a specific response of *V. atrata* to particular ratios of PA and PGPC, different ratio treatments at the same total concentration of compounds were tested. A total concentration of 3 µm was chosen because the sum of the concentrations of PA and PGPC in BCS was close to 3 µm. As shown in Figure 4e, the response of *V. atrata* was dependent on the ratio of PA and PGPC used, with only two ratios (PA: PGPC of 1:2 and 1:4) significantly inhibiting the byssus thread production (*P* < 0.05). Other tested ratios showed no significant effect. It was interesting to note that the molar ratio of PA and PGPC in BCS (1.01: 1.94) is close to 1:2, suggesting that the particular response of *V. atrata* to this ratio of compounds might explain the competitive exclusion observed in the field.

### 2.4 The Effect of PA+PGPC on *V. atrata* Byssus Plaque Properties

**Figure 5:**
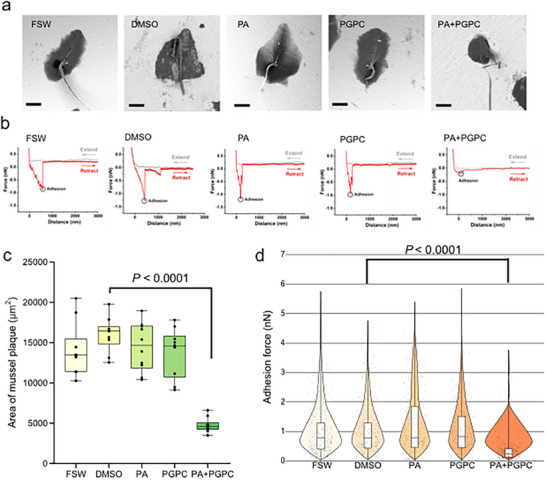
Effect of PA+PGPC on byssus plaque size and adhesive force. For the different treatment groups: a) SEM images of byssus plaques. Scale bar: 50 µm. b) Representative force‐distance curves for DOPA‐mediated adhesion of MFPs in byssal plaques. c) Area of byssal plaque (*n* = 10) and d) Adhesion force of MFPs (*n* > 384). FSW: The FSW control. DMSO: The solvent only control (0.5% DMSO in FSW). PA+PGPC: the mixture of 0.26 µg mL^−1^ PA and 0.96 µg mL^−1^ PGPC. PA and PGPC were tested at concentrations of 0.26 and 0.96 µg mL^−1^, respectively. Significant differences were determined by Dunnett's test. The DMSO group was used as the control for comparison of treatments using Dunnett's test.

Mussels (*V. atrata*) use proteinaceous adhesive plaques to adhere to marine substrates. Here scanning electron microscopy (SEM) was used to examine micromorphology of the adhesive plaque of *V. atrata* under the influence of the mixture of PA and PGPC. As revealed by SEM, exposure to the mixture of 0.26 µg mL^−1^ PA and 0.96 µg mL^−1^ PGPC (as indicated by PA+PGPC in this study) resulted in significantly smaller byssus plaques than those in the DMSO control (**Figure** **5**a), with their area reduced by 70.4% compared to the control. The area of the byssus plaques following PA treatment exhibited no significant difference from the controls, neither did PGPC treatment (Figure 5c). However, it could be seen that PA and PGPC together, synergistically reduced the plaque area of *V. atrata*, suggesting that PA+PGPC may act by inhibiting secretion of MFPs.

Atomic Force Microscopy (AFM) was then used to investigate the effect of PA+PGPC on adhesion strength of the 3,4‐dihydroxyphenylalanine (DOPA) enriched MFPs in the byssus plaques. By analyzing the interaction between the probes and DOPA secreted by *V. atrata* on the petri dish, the adhesion force (defined as the maximum value of the retraction curve) was measured. As indicated by the typical force curves (Figure 5b), MFPs produced under the influence of the PA+PGPC had a lower adhesion force. The violin plot with statistical analysis (Figure 5d) confirmed that the average adhesion force of the PA+PGPC treated MFPs (0.38 ± 0.22 nN) was significantly lower than those deposited under control conditions (0.96 ± 0.56 nN), while PA or PGPC treatment alone showed no effect.

### 2.5 *V. atrata* Gene Expression and PPO Activity Response to PA +PGPC Treatment

**Figure 6:**
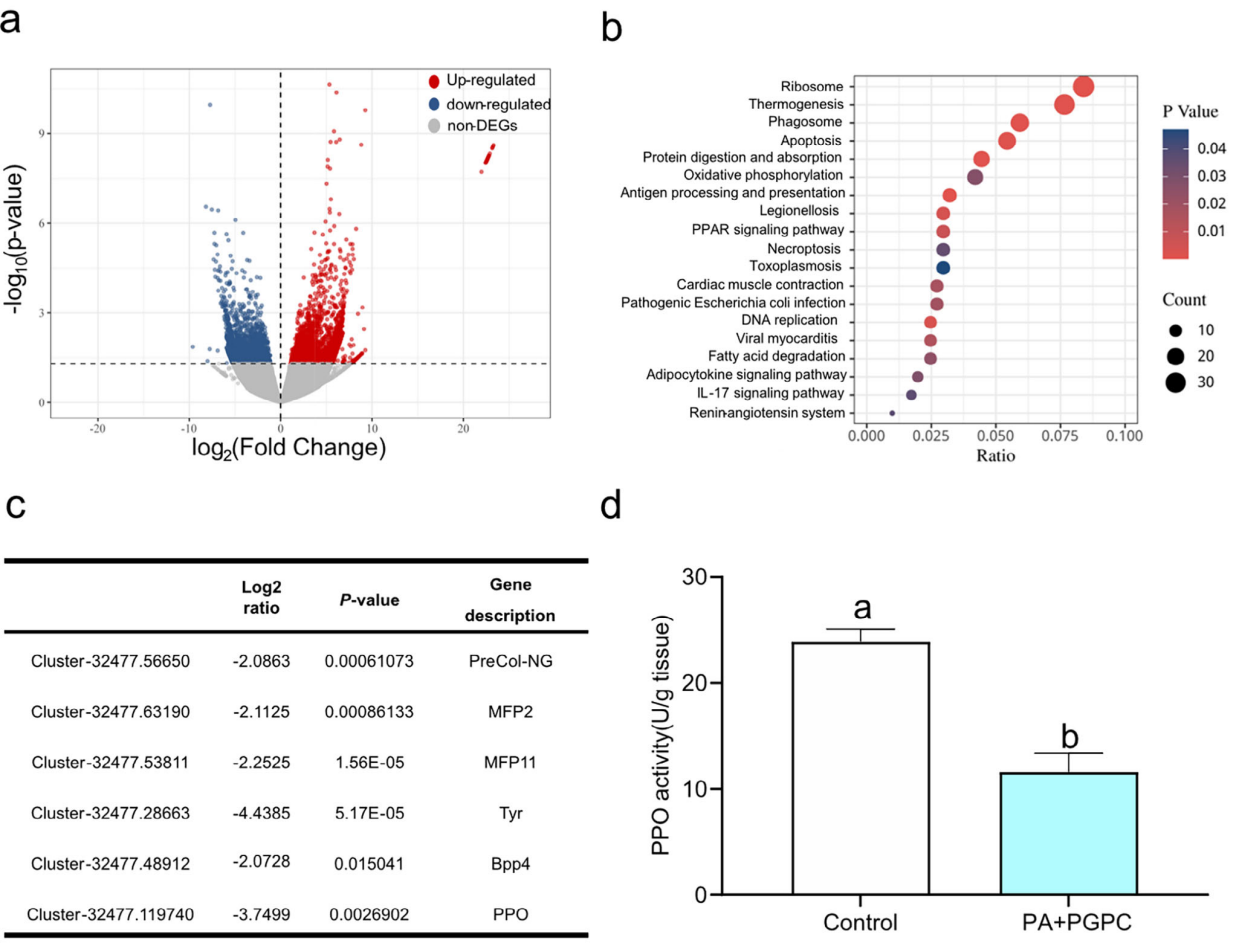
Effect of PA+PGPC on the *V. atrata* transcriptome and PPO activity. a) Volcano plots of DEGs between the control group and the PA+PGPC group. Red and blue spots indicate the up‐regulated and down‐regulated expressed genes, respectively. Grey spots indicate genes that were not differentially expressed. b) KEGG enrichment analysis of DEGs c) List of the key DEGs related to mussel attachment. *PreCol‐NG*: Precollagen‐NG; *MFP2*: Mussel foot protein 2; *MFP11*: Mussel foot protein 11; *Tyr*: Tyrosinase; *BPP4*: Byssal peroxidase‐like protein 4; *PPO*: Polyphenol oxidase. d) Inhibition of PPO activity following PA+PGPC. Data shown are means ± SE of replicates (*n* = 3). Different lower‐case letters above bars represent significant differences (Student's t‐test, *P* < 0.05). Control: The solvent only control (0.5% DMSO in FSW). PA+PGPC: the mixture of 0.26 µg mL^−1^ PA and 0.96 µg mL^−1^ PGPC.

RNA‐seq analysis was used to explore the possible molecular mechanisms underlying PA+PGPC inhibition of *V. atrata* attachment. A summary of sequencing, assembly, and annotation results is shown in Tables S5 and S6 (Supporting Information). All of the RNA‐seq data have been deposited in the NCBI Short Read Archive (SRA) under accession number PRJNA1265923. A total of 4798 differentially expressed genes (DEGs) were identified between the control group and the PA+PGPC group, including 2097 up‐regulated DEGs and 2701 down‐regulated DEGs in the PA+PGPC group compared with the control group (**Figure** **6**a). Kyoto Encyclopedia of Genes and Genomes (KEGG) enrichment analysis of the DEGs suggested that there were 19 pathways significantly enriched (Figure 6b). Notably, the ribosome pathway and the protein digestion and absorption pathway, both related to protein biosynthesis, were significantly enriched (*P *< 0.05; Figure 6b), suggesting that treatment with PA+PGPC influences protein biosynthesis of *V. atrata*. Particular attention was paid to the DEGs related to mussel attachment (Figure 6c). Compared with the control group, treatment with PA+PGPC was found to significantly downregulate the expression level of the genes encoding byssus structural proteins, *PreCol‐NG, MFP2, MFP11*, and those encoding enzymes involved in byssus adhesion (*Tyr, BPP4, and PPO*). These observations may explain why there was a reduction in byssus thread production and reduced attachment. Transcription profiles of these six genes were further validated using quantitative real‐time PCR (qRT‐PCR), the results of which were consistent with those determined by RNA‐seq (Figure S5, Supporting Information).

Polyphenol oxidase (PPO), is a potential enzyme involved in the polymerization and solidification of MFPs.^[^ ^29^ ^]^ As shown in Figure 6d, the activity of PPO after PA+PGPC was significantly lower than that in the control, suggesting that PA+PGPC has an inhibitory effect on the activity of PPO in *V. atrata*. This finding, combined with the above transcriptome results, indicate a possible mechanism for the inhibition of PA+PGPC against *V. atrata* attachment, which includes the down‐regulation of expression of the genes associated with attachment, and decreased activity of the enzyme (PPO) essential for attachment.

## 3 Discussion

### 3.1 Chemical Defense of Calcareous‐Shelled Organisms

We report here the discovery of a chemically‐mediated allelopathic interaction between the two calcareous‐shelled sessile species, *B. albicostatus* and *V. atrata*. Although some studies have demonstrated the use of conspecific cues (or pheromones) in intraspecific aggregation of calcareous‐shelled organisms,^[^ ^30^, ^31^ ^]^ little information is available for the role of allelochemicals in interspecies competition. Barnacles and mussels are both important components of marine benthic ecosystem,^[^ ^32^ ^]^ and are often found to coexist in intertidal zones. It has been reported that in some cases, mussels can gain habitat benefits from barnacles.^[^ ^33^, ^34^ ^]^ Previous research has also suggested a competitive relationship between barnacles and mussels over food and space,^[^ ^35^, ^36^ ^]^ but most of this work looked at the relationship between mussels and their epiphytic barnacles, in which cases mussels were often larger than their epiphytic barnacles. *B. albicostatus* and *V. atrata* however, have similar body sizes. Interestingly, we found that in the field, it is rare for *B. albicostatus* to attach to *V. atrata* individuals and vice versa (Table S7, Supporting Information). Our results provide evidence for the secretion of allelochemicals by *B. albicostatus* which inhibit the attachment of *V. atrata* in the laboratory. It is therefore likely that barnacles are able to use chemical antagonism to defend themselves on the substratum to prevent smothering by mussels. This is also an example of chemical defense being used by barnacles, which are permanently attached in a fixed position, against the more mobile *V. atrata* which can discard their byssus to regain mobility and move away. Ecologically, the reverse was not observed. *V. atrata* could not inhibit cyprid settlement. In contrast, the organisms permanently attached in a fixed position may face greater selective pressure for chemical competition than mobile organisms, including those with limited mobility.

In addition to chemical cues, other factors can influence the recruitment of marine benthic organisms. Mussels may possibly exclude barnacles through physical overgrowth, smothering, or competition for food.^[^ ^35^, ^37^ ^]^ It is commonly assumed that marine calcareous‐shelled organisms lack chemical defense strategies due to their physical protection.^[^ ^38^ ^]^ Our results challenge this view by revealing the presence of allelopathy in these organisms. Our findings therefore call for a broader study of the chemical ecology of other marine calcareous‐shelled organisms such as stony corals, shipworms, and oysters to deepen our understanding of marine ecosystem dynamics.

### 3.2 Synergistic Allelopathy Among Marine Animals: A New Paradigm

Combining chemical and biological analytical approaches, we found that PA and PGPC released from *B. albicostatus* can act as allelochemicals, which can synergistically inhibit *V. atrata* attachment. Both PA and PGPC are common metabolites.^[^ ^39^, ^40^ ^]^ In a previous study exploring natural antifouling products as antifoulants, PA was isolated from the sponge *Phyllospongia papyracea* which was active in inhibiting attachment of the mussel *Mytilus edulis*.^[^ ^41^ ^]^ Here the synergistic effect of a precise mixture of PA and PGPC in inhibiting mussel attachment was observed. Secreting such mixtures of compounds, may play an important ecological role in providing barnacles with a competitive advantage in intertidal communities and explain their global success as hard substrate colonisers. The use of a blend of different infochemical components with a synergistic effect has also been found in pheromones of insects^[^ ^42^ ^]^ and mussels^[^ ^30^ ^]^ and in defense cues of terrestrial plants against herbivores,^[^ ^43^ ^]^ but whether marine invertebrates use synergistic infochemical mixtures for space competition remains poorly understood.

Our work adds a rare marine perspective to the predominantly terrestrial‐focused literature on synergistic allelopathy, particularly among invertebrates with calcareous shells. Employing common metabolites (PA and PGPC) as allelochemicals, instead of lineage‐specific toxins, may reflect an evolutionary adaptation to minimize investment of metabolic energy in this important function. Future research should examine whether this mechanism is widespread among marine sessile invertebrates. It is also fascinating that only particular molar ratios of PA and PGPC (1:2 and 1:4) could inhibit byssus thread production of *V. atrata*. This suggests that there may be species‐specific responses based on the ratio of the different components. Interestingly, *B. albicostatus* releases PA and PGPC into seawater with the molar ratio ≈ 1:2. In the marine environment, *V. atrata* may have evolved an avoidance response to this specific ratio of PA and PGPC from neighboring *B. albicostatus* by altering their attachment behavior.

It should be noted that for chemical cues to function in marine environments they should be present in the field at ecologically relevant concentrations to exert an effect on the receiving organisms.^[^ ^3^, ^44^ ^]^ PA and PGPC could be released from *B. albicostatus* along with MCF into the surrounding water during feeding and reproductive activity when the barnacle operculum is opened. In the field, during mass feeding (and/or reproduction) of densely‐aggregated barnacles, adjacent *V. atrata* likely experience ecologically relevant concentrations of the synergistic mixture of PA and PGPC. Future work is needed to investigate the in situ concentration range of these allelochemicals experienced by *V. atrata* to better understand the chemically‐mediated competition between these two species.

### 3.3 Molecular Mechanism of Inhibition Against Mussel Attachment

**Figure 7:**
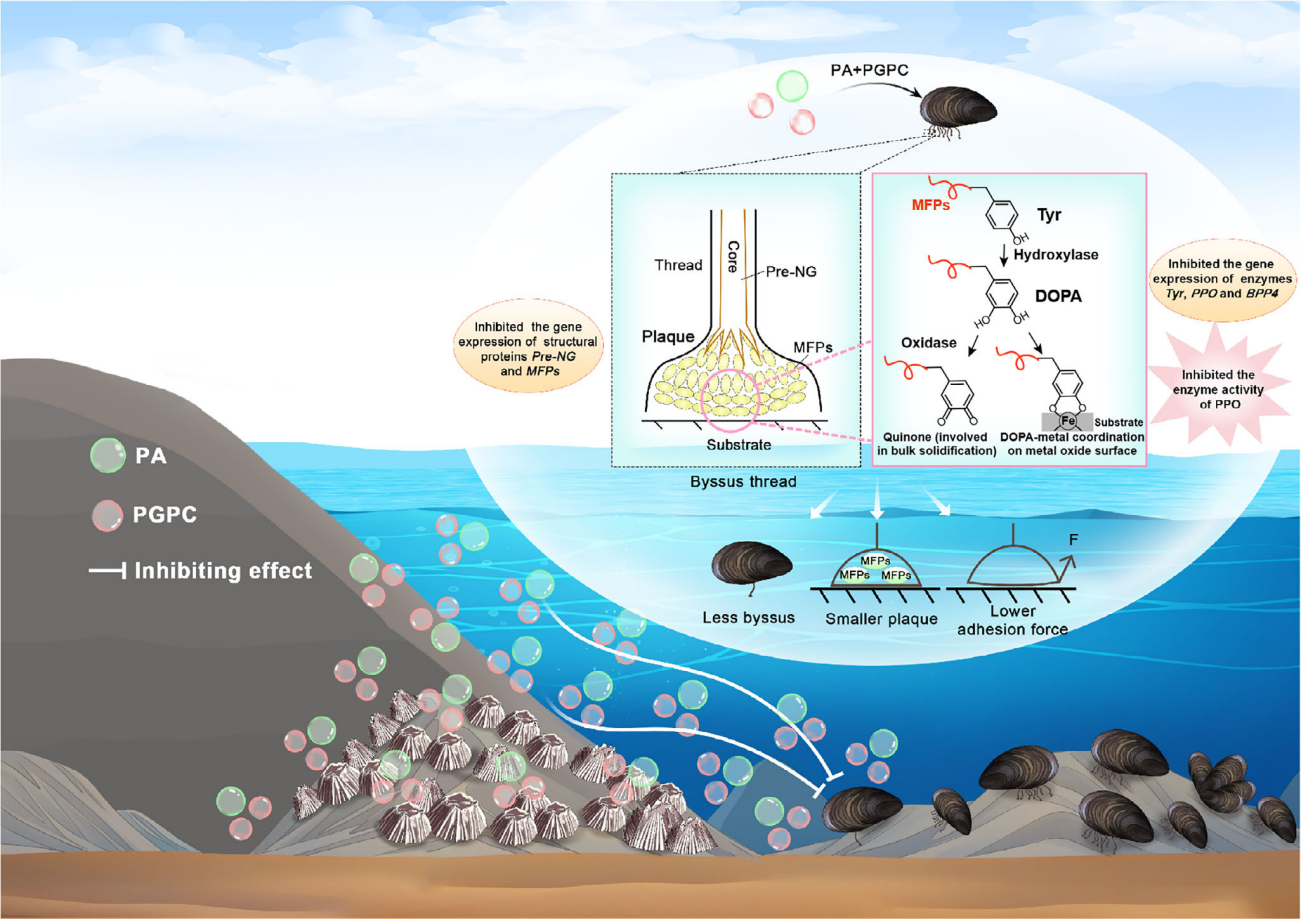
Two common metabolites synergistically mediate spatial competition between barnacles (*B. albicostatus*) and mussels (*V. atrata*). *B. albicostatus* releases a synergistic blend of PA and PGPC at a ratio of ≈ 1:2 as the allelochemical which inhibits the attachment of *V. atrata*, allowing the barnacle to gain a competitive advantage in defending its position on the substratum in the intertidal zones. Exposure to the mixture of PA and PGPC down‐regulated the expression of the genes associated with mussel byssus formation and adhesion, and decreases the activity of an enzyme (PPO) essential for mussel adhesion. As a result, *V. atrata* produced fewer byssi and with smaller and weaker adhesive plaques. Note that the partition between barnacles and mussels in this schematic figure is to emphasize the chemical defense of *B. albicostatus* against *V. atrata*. In the field, these two species can co‐occur in intertidal habitats with an inverse relationship between their densities as shown in Figure 1.

Strong adhesion on surfaces is critical for mussel survival in the marine environment, particularly in tidal areas. MFPs have received attention not only in adhesion science but also in biomimetic applications such as medical adhesives, mainly due to their ability to adhere to wet surfaces and their biocompatibility.^[^ ^45^ ^]^ Here, we found that mussels exposed to the blend of PA and PGPC secreted significantly less byssus and smaller plaques, suggesting that the production of proteins involved in the formation of byssal threads and plaques is inhibited. This was supported by the results of the transcriptomic analysis, which indicated that PA+PGPC impacts protein biosynthesis of *V. atrata* by influencing the ribosome pathway and protein digestion and absorption pathways. Furthermore, expression of the genes associated with byssus formation and adhesion (*PreCol‐NG*, *MFP2*, *MFP11, Tyr*, *BPP4* and *PPO*) were significantly down‐regulated by PA+PGPC. PreCol‐NG is an important collagen protein contributing to the formation of the inner fibrous core of mussel byssus.^[^ ^46^ ^]^ The mussel foot proteins MFP2 and MFP11 are distributed throughout the mussel adhesive plaque, contributing to the structural integrity of the plaque and its adhesive properties.^[^ ^47^, ^48^, ^49^ ^]^ The three enzymes Tyr, BPP4 and PPO are involved in hydroxylation and/or oxidation reactions, which are important for byssal adhesion.^[^ ^29^, ^50^, ^51^ ^]^ In particular, Tyr and PPO have been suggested to occur in mussel byssus.^[^ ^50^, ^52^ ^]^ MFPs are well‐known for post‐translational modification, with tyrosine residues being hydroxylated to DOPA.^[^ ^53^, ^54^ ^]^ DOPA is critical for adhesion of MFPs to substrates, mainly through hydrogen bonding, aromatic interactions and metal coordination.^[^ ^55^ ^]^ DOPA‐metal coordination also contributes to the mechanical properties of mussel byssus.^[^ ^56^ ^]^ Furthermore, it is the prevailing view that oxidative enzymes such as PPO play an essential role in catalysis of DOPA to o‐quinone, which takes part in MFP cross‐linking reactions and leads to the solidification of MFPs.^[^ ^57^ ^]^ Additional experimental evidence is needed to confirm the precise role of these enzymes in byssus formation. The finding that PA+PGPC inhibited PPO activity suggested that the polymerization and solidification of MFPs might be impaired by PA+PGPC, thus impacting cohesion of MFPs. Taken together, the reduced number of byssi, smaller plaques and the lower adhesion forces observed following PA+PGPC of *V. atrata* may be caused (or partly caused) by reduced expression of the genes encoding structural proteins (*PreCol‐NG*, *MFP2*, *MFP11*) and essential enzymes (*Tyr*, *BPP4* and *PPO*) and the decreased activity of PPO (**Figure** **7**). For mussels, the inhibited attachment may impair their ability to withstand high shear forces from waves and other stressors in the intertidal zone. Interestingly, PA and PGPC can affect gene expression in fish and mice possibly due to the indirect effect of these two compounds on transcription factors.^[^ ^58^, ^59^ ^]^ Here, we speculate that PA and PGPC may similarly inhibit gene expression in mussels via transcription factors.

### 3.4 Harnessing Allelopathy for Sustainable Antifouling

Our findings also provide important insights for the development of biofouling control. Marine fouling organisms such as barnacles and mussels often settle on submerged structures, which causes serious fouling problems affecting many industries.^[^ ^20^, ^60^ ^]^ Fouling reduces ship speed, increases fuel cost and emission of greenhouse gases, and leads to the spread of invasive aquatic species.^[^ ^20^ ^]^ It also negatively affects aquaculture nets, underwater sensors, marine platforms, etc. Metal‐based antifoulants (such as copper and tributyltin) and synthetic organic biocides (such as cybutryne and diuron) have been widely used to prevent biofouling. However, their toxicity and adverse environmental impacts have led to bans and restrictions on their use.^[^ ^61^, ^62^, ^63^ ^]^ Consequently, the growing stringency of environmental regulations has rendered the development of environmentally friendly alternatives a critical priority. Our finding that the two biodegradable natural products PA and PGPC have reversible inhibitory activity against mussel attachment, suggests their potential as green antifoulants. More importantly, the finding that the allelochemicals exhibit a synergistic effect in a precise ratio at low doses, suggests the promising use of a synergistic combination of antifoulants in biofouling control, which may significantly reduce the amount and cost of antifoulants used in practical applications.

## 4 Conclusion

In summary, through an interdisciplinary approach integrating ecological, chemical, and molecular biological analysis, our study reveals that synergistic allelopathy of common metabolites serves as a cryptic driver of intertidal competition patterns. The barnacle *B. albicostatus* deploys a synergistic blend of PA and PGPC to inhibit attachment of the mussel *V. atrata*. The underlying action mechanism of allelochemicals involves down‐regulating the expression of key genes and decreasing enzymatic activity vital for mussel byssus formation and adhesion. These findings offer valuable insights into intertidal community interactions and can inspire the development of effective marine antifouling strategies.

## 5 Experimental Section

### Field Survey

The quantitative field survey was conducted in September 2021. Three study sites were selected along the coast of Xiamen in southeastern China, i.e., Wuyuan Bay (N24°52′, E118°16′), Baicheng Bay (N24°43′, E118°10′), and Haicang Bay (N24°47′, E117°98′) (Figure 1a–c). At each site, three replicates of the sampling zones with the same tidal heights were established, with a distance of ≈ 20 m between each. In each replicate sampling zone, the subzones with high, medium and low densities of *B. albicostatus* were chosen. In each subzone, a 20 cm x 20 cm wooden frame was used, and numbers of *B. albicostatus* and *V. atrata* were counted (Figure S1, Supporting Information).

### The Effect of VCS on Settlement of *B. albicostatus*

Preparation of VCS was shown in Supporting Information. Here *B. albicostatus* cyprid larvae obtained as described in were used for the settlement assay.^[^ ^64^ ^]^ Briefly, field‐collected adults of *B. albicostatus* were dried overnight in the lab and then immersed in seawater, after which they released nauplii. The nauplii were cultured in FSW at 28 °C at a density of 1 larva mL^−1^ and fed daily with the microalga *Platymonas subcordiformis* at 1 × 10^5^ cells mL^−1^. After 4‐5 days of incubation, most nauplii had metamorphosed to cyprids. The cyprids were then harvested by filtration with a 200 µm mesh size plankton net and stored at 4 °C for one day before being used in assays. The single‐larva assay was performed to avoid possible inductive effects of the conspecifics.^[^ ^65^, ^66^ ^]^ Each replicate consisted of a 24‐well plate containing 20 cyprids (one per well), with 1 mL of VCS added into each well. After incubating in the dark for 24 h at 26 °C, the cyprid in each well was observed under an inverted microscope (Leica DMIL, German). The total number of cyprids that had settled in the 20 wells of each plate was counted, and the proportion of settlement (out of 20 larvae) was calculated for each plate (i.e., each replicate). There were three replicate plates (*n* = 3). FSW was used as the control.

### The Effect of BCS on Attachment of *V. atrata*

Preparation of BCS was shown in Supporting Information. The effect of BCS on the attachment of *V. atrata* was tested following the method described previously^[^ ^67^ ^]^ with some minor modifications. The byssus of *V. atrata* was gently cut off with scissors. Each replicate consisted of a 24‐well plate containing 12 mussels (one per well), with 1 mL of BCS added into each well. After culturing in the dark for 24 h at 26 °C, the total number of byssus threads produced by the 12 mussels in each plate was counted, and the average number of byssus threads per mussel was calculated for each plate (i.e., each replicate). There were three replicate plates (*n* = 3). FSW was used as the control.

### Chemical Analysis of MCF of *B. albicostatus*

The above assay on the effect of BCS suggested that *B. albicostatus* release chemical cues which inhibit byssus production of *V. atrata* (see results). Barnacles can release substances from their MCF when the operculum opens. Therefore, chemical analysis of *B. albicostatus* MCF was performed. To collect MCF, the adults of *B. albicostatus* (0.6–1.3 cm in base length, 0.4–1.1 cm in base width, 0.4–1.0 cm in height) were placed on a table and their opercula were carefully broken open with a screwdriver. Then MCF was collected using an Eppendorf pipette. A total of 20 mL MCF was collected from ≈ 200 *B. albicostatus* adults. The MCF was centrifuged (6500 rpm, 4 °C, 20 min) and filtered (0.22 µm), after which it was analyzed by LC‐MS/MS (Supporting Information).

### The Effect of Metabolites from MCF on Attachment of *V. atrata*

There were 181 compounds identified using the above LC‐MS/MS analysis (see results). From these 181 compounds detected in the MCF of *B. albicostatus*, compounds were selected that could be purchased commercially and ranked them by relative abundance in MCF from high to low. The top 35 compounds (Table S5, Supporting Information) were examined here. They were purchased from Sigma–Aldrich Co., Ltd. (St. Louis, MO, USA), SHANGHAI ZZBIO CO., Ltd. (Shanghai, China) and Aladdin Reagent CO., Ltd. (Shanghai, China). Lipophilic compounds were dissolved in DMSO, and the hydrophilic compounds dissolved in FSW. For each lipophilic compound, one mussel, 5 µL of each treatment, and 995 µL of FSW were added into each well of a 24‐well plate. For each hydrophilic compound, one mussel and 1 mL of treatment solution were added into each well. Here the test concentration for each compound was set at 50 µg mL^−1^ to screen out potential inhibitory active compounds against mussel attachment. The FSW control and the solvent control (0.5% DMSO in FSW, V/V) were both set up in this assay. There were three replicates for each treatment. Each replicate included 12 mussels, with one mussel in one well. After 24 h in the dark, the number of byssus threads produced by each mussel was counted.

Of the 35 tested compounds, four compounds were found to be active in inhibiting *V. atrata* attachment at 50 µg mL^−1^ and were further investigated to determine EC_50_ values (the concentration that inhibited byssal thread production of mussel by 50% relative to the control) and the residual effect. The inhibitory compounds were PA, MGPC, PGPC, and NET. The effects of 24 h exposure of *V. atrata* to different concentrations of these four compounds were tested as described above, with the tested concentrations between 1 and 100 µg mL^−1^. To test whether there was a residual effect, after the first 24 h exposure period, the attached mussels were gently cut off their byssus using scissors, washed with FSW and transferred into the new 24‐well plates. Similarly, the unattached mussels were washed with FSW and also transferred into the new 24‐well plates. Each well in the new 24‐well plates contained 1 mL FSW and one mussel. After post‐exposure cultivation for a further 24 h, the number of byssus threads produced by each mussel was counted.

### Determination of the Concentrations of PA and PGPC in *B. albicostatus* BCS and their Synergistic Effect on *V. atrata* Byssus Production

The presence and the concentrations of the above four active metabolites (PA, MGPC, PGPC, and NET) in B. albicostatus BCS were examined by Q‐Exactive Orbitrap mass spectrometry (Thermo, USA) coupled with an Ultimate 3000 HPLC system (Thermo, USA). The details for LC‐MS analysis were shown in Supporting Information. The results were analyzed using Xcalibur 4.0 software to calculate concentrations. Standard curves of these four standard compounds were generated by plotting the extracted ion chromatogram (EIC) peak area versus the five concentrations (0.01, 0.05, 0.1, 0.5, and 1 µg mL^−1^). Quantification of these compounds in BCS was achieved based on regression analysis of peak area against concentration.

It was found that PA and PGPC are present in *B. albicostatus* BCS at concentrations lower than the lowest inhibitory concentrations measured in the assays. It was therefore hypothesized that there may be a synergistic effect between PA and PGPC, which was investigated as follows. The byssus thread production of *V. atrata* in response to the individual compounds at their respective concentrations in BCS (0.26 µg mL^−1^ for PA and 0.96 µg mL^−1^ for PGPC) and their mixture (0.26 µg mL^−1^ PA + 0.96 µg mL^−1^ PGPC, as indicated by the SE treatment in this study) were tested as described above. FSW and 0.5% DMSO (in FSW, V/V) were used as negative controls. BCS was used as the positive control.

To further test whether the ratio of PA and PGPC would influence the response of *V. atrata*, the effects of different ratios of PA and PGPC were investigated. Here 9 treatments with different molar ratios of PA: PGPC (1:0, 1:1, 1:2, 1:4, 1:8, 0:1, 2:1, 4:1, 8:1) were set up. The total concentrations of PA and PGPC in each treatment was the same, which was 3 µm, since the sum of concentrations of these two compounds in BCS was close to 3 µm. Byssus thread production of *V. atrata* in response to each treatment was tested as described.

### The Synergistic Effect of PA and PGPC on the Adhesive Plaque of *V. atrata*

Here SEM (Phenom Prox, Bruker, Germany) was used to examine micromorphology of the adhesive plaque of *V. atrata* under the influence of the mixture of PA and PGPC. In each well of a 24‐well plate, a piece of aluminum foil was placed over the surface of well bottom and sides. Then one mussel and 1 mL treatment solution were added into each well. PA and PGPC were tested respectively at 0.26 and 0.96 µg mL^−1^ (i.e., their concentrations in BCS), and their mixture (0.26 µg mL^−1^ PA + 0.96 µg mL^−1^ PGPC, PA+PGPC) was also tested. FSW and 0.5% DMSO (in FSW, V/V) were used as controls. There were three replicates for each treatment. Each replicate included 12 mussels, with one mussel in one well. After culturing in the dark for 24 h, the aluminum foil from the wells, with byssus plaques, was cut into 1 cm × 1 cm pieces and placed in 1.5 mL centrifuge tubes. The samples were washed with physiological saline to clean the byssus surface. Then the samples were placed in 4% paraformaldehyde fixative and kept at 4 °C overnight for fixation. After that, the samples were washed three times with 0.1 m phosphate buffer for 20 min each. Dehydration was performed in a gradient of ethanol at 30% and 50% for 5 min each, followed by 10 min each at 70% and 80% ethanol, and finally 15 min each at 95% and 100% ethanol (with anhydrous sodium sulfate treatment). The critical point drying method (CPD, Leica CPD300, German) was immediately used on samples for 2 h. After drying, the samples were adhered to conductive tape and gold‐coated for 60 s before being observed and photographed under a SEM (Phenom Prox, Bruker, Germany). Vacuum mode was used and photographs were taken at a magnification of 800x. Images were analyzed using Adobe Photoshop 2022 to calculate the area of the adhesive plaques in different treatments.

The effect of PA+PGPC on adhesion of plaque proteins was also examined by AFM (JPK NanoWizard 4 XP, Bruker, German) equipped with an inverted microscope (Eclipse Ti2, Nikon, Japan). The treatments of PA, PGPC, PA+PGPC and the controls were set up as described above. For each treatment, three mussels and 5 mL of treatment solution were placed in a 35‐mm‐diameter petri dish and incubated in the dark for 3 h, during which the mussels produced byssus plaques. The byssus was then gently cut with sterilized scissors and the solution was removed from each petri dish, followed by the addition of 2 mL FSW. The protein–surface adhesion was assessed by following the methods of Zhong et al. (2014) and Guo et al. (2014) with minor modification.^[^ ^68^, ^69^ ^]^ In this study, a conical probe (NanoWorld PNP‐TR‐20‐ CantileverA) was employed in contact mode (Contact Mode Force Spectroscopy). After calibration, the probe's deflection sensitivity (Z sense) was 8.09 nm V^−1^ and the spring constant of the cantilever (K) was 0.269 N m^−1^. During scanning, the AFM tip made multiple contacts with plaque proteins on the surface, with controlled loading parameters (1.5 nN setpoint, 6 µm s^−1^ retraction speed, 0.5 s contact time) to form adhesive interactions between the tip and plaque proteins transiently and maintain an appropriate measurement speed for high‐quality force curves. During scanning, the tip did not punctuate the plaque matrix (i.e., non‐destructive contact), as evidenced by the absence of substrate‐contact signatures in the force curves (Figure 5b). During retraction, the protein–tip interactions were progressively unloaded until detachment occurred. This process typically produced force curve with multiple peaks, reflecting sequential unfolding events of the MFPs followed by their final detachment from the tip surface, thereby reflecting their adhesion. Therefore, the maximum rupture force observed in each curve was taken as the adhesion force between MFPs and the AFM tip. Three random areas (10 µm × 10 µm) within the byssus plaque were selected, with a force resolution set at 15 × 15, resulting in 225 force curves obtained from each area.

### Transcriptome Analysis of the Effect of PA+PGPC on *V. atrata*

Gene expression profiles of *V. atrata* were explored in two groups: the control group (individuals of *V. atrata* exposed to FSW with 0.5% DMSO for 24 h), and the PA+PGPC group (individuals of *V. atrata* exposed to PA+PGPC dissolved in FSW with 0.5% DMSO for 24 h). Each sample group had three replicates, with each replicate containing feet from ten mussels (Due to the tiny foot of *V. atrata* and to ensure a sufficient quantity of RNA for transcriptome analysis, feet from ten mussels for each replicate) was collected. The foot of *V. atrata* was dissected, cooled in liquid nitrogen for 20 min, and subsequently stored on dry ice. The RNA‐seq was performed by Novogene Bioinformatics Technology Co., Ltd. (Beijing, China). The total RNA was extracted from mussel feet using the Trizol method, and the quality of RNAs was assessed using an Agilent 2100 Bioanalyzer system (Agilent Technologies, Santa Clara, CA, USA). First, a total RNA library was constructed and quantified using the Agilent 2100 Bioanalyzer system to ensure the quality of the library, followed by sequencing using the Illumina HiSeq NovaSeq 6000 system. To ensure reliability, raw data was filtered. Clean reads were obtained by removing reads containing adapters, N bases, and low‐quality reads from the raw data. Meanwhile, the Q20, Q30, and GC content of the clean data were also calculated. Clean reads were assembled using Trinity v2.4.0 software,^[^ ^70^ ^]^ the transcriptome assembled by Trinity was used as the reference sequence, and RSEM software was employed to map the clean reads of each sample. Differential gene expression was calculated using the Fragments Per Kilobase per Million reads (FPKM) method. Gene function annotation was performed based on the Nr, Nt, Pfam, KOG/COG, Swiss‐Prot, KEGG, and GO databases. The DESeq2 R package (version 1.20.0) was employed to analyze DEGs across different groups. Genes with P‐value ≤ 0.05 and the absolute value of log2 (fold change) ≥ 1 were considered differentially expressed. GO enrichment analysis and KEGG pathway analysis of DEGs were implemented by the clusterProfiler R package. DEGs were considered significantly enriched when the P‐value was ≤ 0.05. To verify the transcriptome results, qRT‐PCR analysis was performed for 6 DEGs (Figure S5, Supporting Information). These were *PreCol‐NG*, *MFP2*, *MFP11, Tyr*, *BPP4*, and *PPO* and are involved in mussel byssus formation and adhesion.^[^ ^29^, ^46^, ^47^, ^48^, ^50^, ^51^ ^]^ The qPCR primers were designed using Primer 3.0 (Table S8, Supporting Information). The details of qRT‐PCR analysis are shown in Supporting Information.

### The Effect of PA+PGPC on the Activity of PPO of *V. atrata*

PPO is an essential enzyme involved in mussel attachment. *V. atrata* was cultured in the PA+PGPC (PA+PGPC dissolved in FSW with 0.5% DMSO) for 24 h. *V. atrata* cultured in FSW with 0.5% DMSO only was used as the control. Each group had three replicates, with each replicate containing ten mussels. After 24 h of culture, the foot of *V. atrata* was obtained by dissection (each group containing feet from ten mussels), cooled in liquid nitrogen for 20 min, and subsequently stored at −80 °C. PPO activity was measured using a PPO activity kit (Nanjing Jiancheng Bioengineering Institute, China), with enzyme activity expressed in U/g. PPO can catalyze phenol to quinone, which exhibits characteristic absorption at 420 nm. The OD_420_ of each solution was measured using a Multimode Reader (Infinite M200 Pro, Tecan, Switzerland).

### Statistical Analysis

Results were analyzed with SPSS 26.0 software. For the assays of compounds on mussel byssus thread production and the assays of PA+PGPC on byssus plaque, one‐way analysis of variance (ANOVA) was performed with a Dunnett's post‐hoc test for multiple comparisons of treatment means with the control. For the assays testing the activities of BCS and VCS, the qRT‐PCR assay and the assay of PA+PGPC on PPO activity, the Student's t‐test was used to calculate the statistical significance between two data groups. Pearson's correlation coefficient for the field survey data was calculated using Excel 2018. The EC_50_ of compounds against mussel byssus production was calculated using GraphPad Prism 8.4. The significance level was defined as *P* < 0.05.

## Conflict of Interest

The authors declare no conflict of interest.

## Supporting information



Supporting Information

## Data Availability

The data that support the findings of this study are available from the corresponding author upon reasonable request.
