# Supplementary material for: Intertidal Warfare: Synergistic Allelopathy Mediates Spatial Competition between Two Marine Calcareous‐Shelled Sessile Organisms
Source: Adv Sci (Weinh). 2025 Dec 7;13(11):e12644. doi: 10.1002/advs.202512644 (PMC12931170; doi:10.1002/advs.202512644)
Supplement: Supplementary file 1 — Supporting Information [file ADVS-13-e12644-s001.docx]

**Supporting Information**

Supporting Information is available from the Wiley Online Library or from the author.

Supporting Information

Intertidal Warfare: Synergistic Allelopathy Mediates Spatial Competition Between Two Marine Calcareous-Shelled Sessile Organisms

Zhuo Li, Zixin Huo, Shanshan Yao, Xianmeng Liang, Yiran Zhao, Yanxin Wang, Shifeng Guo, Caihuan Ke, Pei Su, Danqing Feng^*^

**This additional file includes:**

Supplementary Text

Figures S1-5

Tables S1-8

Supplementary Text

**Preparation of *V. atrata* conditioned seawater (VCS)**

Individuals of *V. atrata* were collected from Wuyuan Bay using sterilized scissors to carefully cut the byssal threads. Adults (0.8-1.4 cm long, 0.3-0.7 cm wide and 0.2-0.4 cm in height) were cleaned with a toothbrush, then rinsed with 0.22-μm-filtered seawater (FSW). VCS was prepared by placing 100 individuals in 1 L FSW for 12 h at 28 °C and filtering (0.22 μm) prior to use.

**Preparation of *B. albicostatus* conditioned seawater (BCS)**

*B. albicostatus* individuals were also collected from Wuyuan Bay. Adults (0.6-1.3 cm in base length, 0.4-1.1 cm in base width, 0.4-1.0 cm in height) were cleaned using a toothbrush and rinsed with 0.22-μm-filtered seawater (FSW). BCS was prepared by placing 25 individuals into FSW (250 mL) for 12 h at 28 °C. The resulting liquid was then filtered (0.22 μm).

**LC-MS/MS analysis of MCF of *B. albicostatus***

UHPLC (1290 Infinity LC, Agilent Technologies) coupled to a quadrupole time-of-flight mass spectrometer (AB Sciex TripleTOF 6600) was used in this study. For liquid chromatography, the Agilent 1290 Infinity LC UHPLC system with a HILIC column was employed for the precise separation of samples. During the entire chromatographic analysis, the column temperature was maintained at a constant 25 °C with a flow rate set to 0.5 ml min^-1^ and an injection volume of 2 μL per sample. Two compositions were selected for the mobile phase: A phase consisted of a mixture of water with 25 mM ammonium acetate and 25 mM ammonia solution, while B phase was pure acetonitrile. The specific steps of the gradient elution program were as follows: from 0 to 0.5 min, 95% B; from 0.5 to 7 min, B linearly decreased from 95% to 65%; from 7 to 8 min, B linearly decreased from 65% to 40%; from 8 to 9 min, B was maintained at 40%; from 9 to 9.1 min, B linearly increased from 40% to 95%; and finally, from 9.1 to 12 min, B was maintained at 95%. After the UHPLC analysis, the samples were subjected to MS analysis in both positive and negative ionization modes. The conditions were as follows: ion Source Gas1 as 60, Ion Source Gas2 as 60, curtain gas as 30, source temperature: 600°C, IonSpray Voltage Floating as ± 5500 V, TOF MS scan m/z range as 60 to 1000 D, product ion scan m/z range as 25 to 1000 D and collision energy as 35 ± 15 eV.

**LC-MS analysis of *B. albicostatus* BCS for determination of the concentrations of active metabolites**

The presence and the concentrations of the four active metabolites (PA, MGPC, PGPC and NET) in *B. albicostatus* BCS were examined by LC-MS. The methods of LC-MS were as follows: pure methanol and 10 mM ammonium acetate were mobile phases B and A. A total of 20 μL of BCS was separated on a C18 SB-AQ, 250 mm × 4.6 mm, 5 μm, chromatography column. BCS was eluted with a solvent composition beginning with 98:2 (A: B) for 10 min and kept to a gradient of 20:80 in 20 min, and ultimately 10 min at 20:80. The flow rate was 1.0 mL/min and the column temperature was 30 °C, the UV wavelength was 254 nm. The conditions of MS analysis in both positive and negative ionization modes were as follows: the IonSpray voltage set to ± 3800 V, with sheath gas flow rate at 40 arbitrary units, the auxiliary gas flow rate at 10 arbitrary units, the probe heater temperature at 350 °C, the capillary temperature at 320 °C, product ion scan m/z range at 100 to 750.

**Validation of differentially expressed genes by qRT-PCR**

To verify the transcriptome results, qRT-PCR analysis was performed for 6 DEGs (Supplementary Figure 2). Total RNA was extracted using the TRIzol® reagent, followed by reverse transcription (Takara, Japan) to obtain cDNA. The RT-qPCR was performed on an Applied Biosystems 7500 Real-Time PCR System (QuantStudio 6 Flex, ABI, USA) using FastStart Universal SYBR Green Master (Sigma-Aldrich, Germany) according to the following protocol: 95°C for 10 minutes, 15 seconds at 95°C, and 1 minute at 60°C for 40 cycles. All reactions were carried out in a final volume of 20 μL, with cDNA from *V. atrata* serving as the template. Each sample was analysed in triplicate. The 2^-ΔΔCt^ method was used to assess the relative expression levels of DEGs. The reference gene used for relative expression calculation was β-actin.


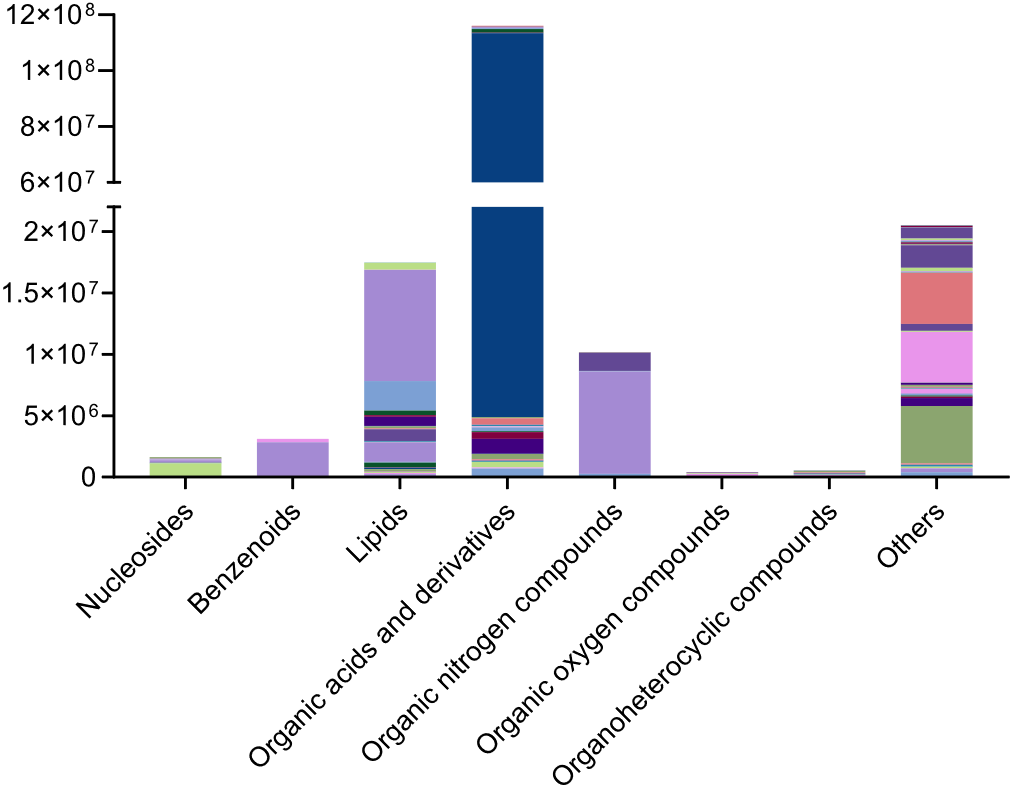


Figure S1. Relative abundance of compounds in the metabolomes of MCF. Derived from replicate 20 mL samples (n = 200). Colors represent the chemical superclass.


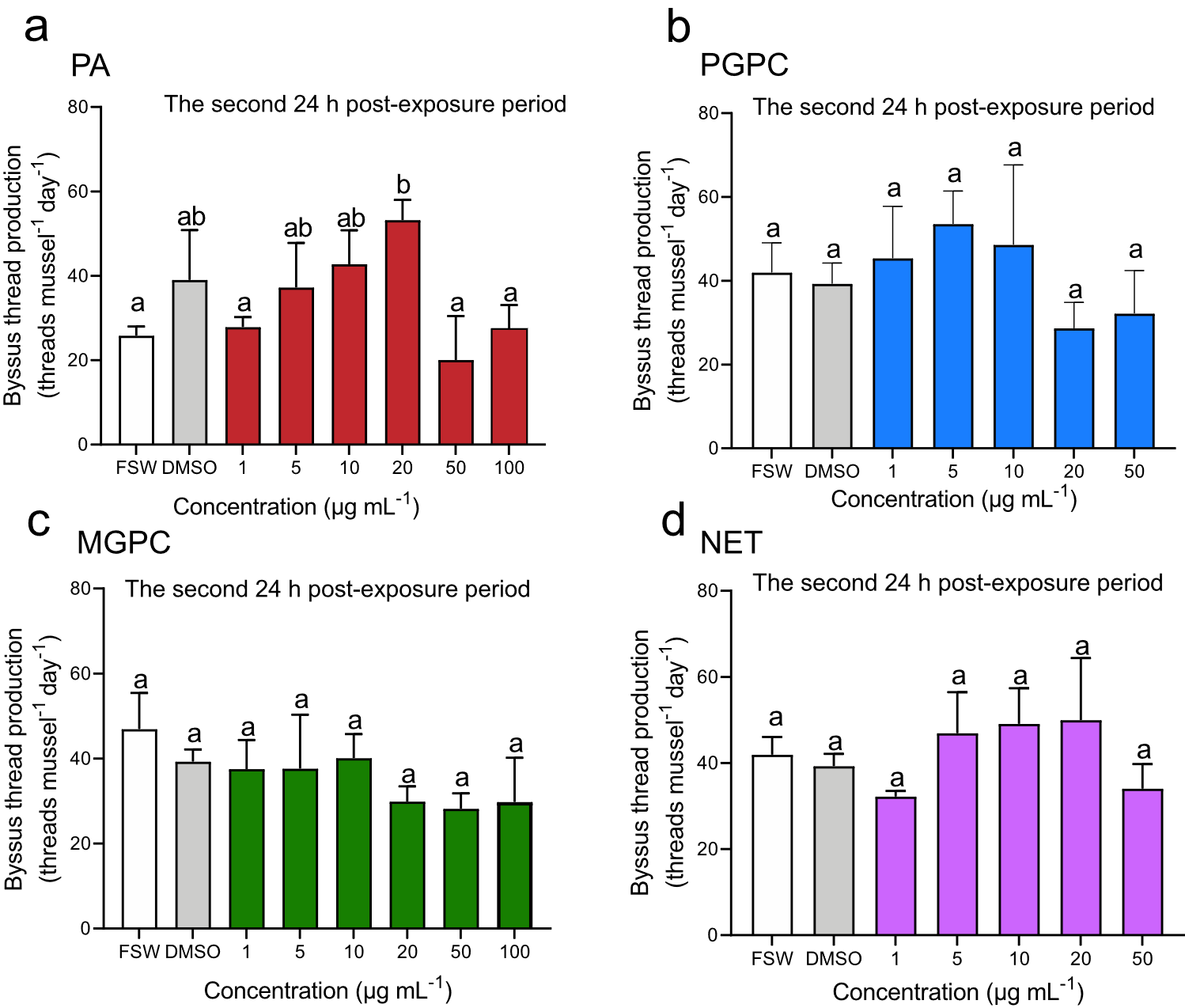


Figure S2. Byssus thread production by *V. atrata* after exposure to PA, MGPC, PGPC and NET for a 24 h period and then in the absence of these compounds in the subsequent 24 h post-exposure period (residual effect). The result of the first 24 h exposure is shown in Figure 3d-g. FSW: The filtered seawater control. DMSO: The solvent only control (0.5% dimethyl sulfoxide in FSW). Data shown are means ± SE of replicates (n = 3). Different lower-case letters above bars represent significant differences (Dunnett’s test, *P* <0.05). The DMSO group was used as the control for comparison of treatments using Dunnett’s test.


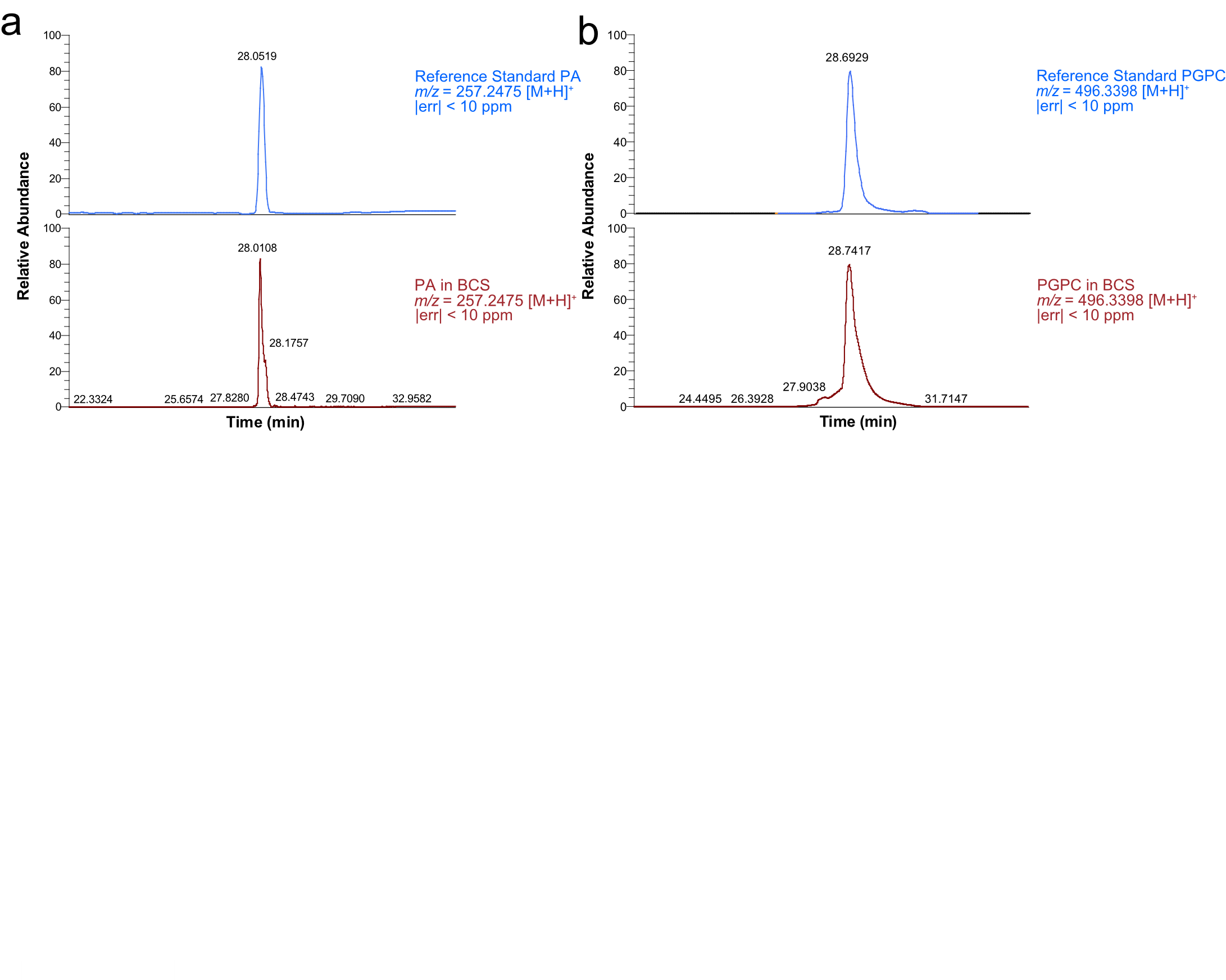


Figure S3. Determination of the presence of PA and PGPC in *B. albicostatus* BCS by LC-MS. a) EIC of PA from the reference standard and BCS. b) EIC of PGPC from the reference standard and BCS.


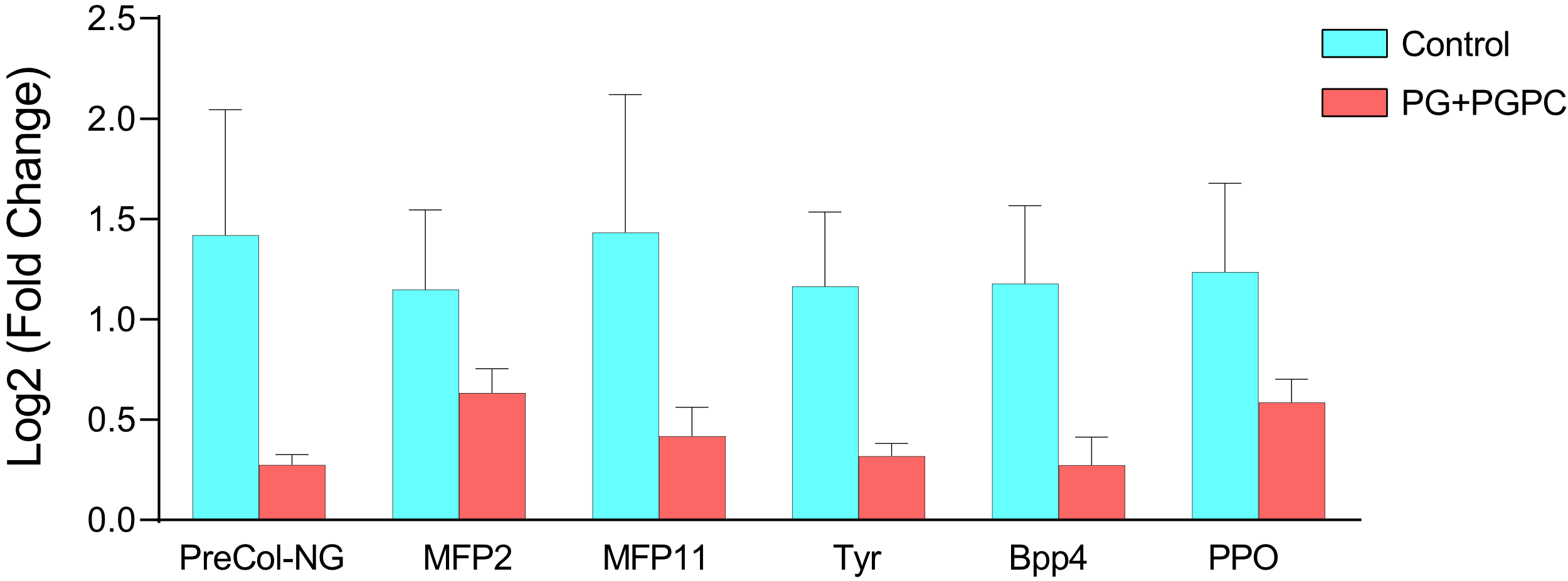


Figure S4. qRT-PCR results of key DEGs related to mussel attachement. PreCol-NG: Precollagen-NG; MFP2: Mussel foot protein 2; MFP11: Mussel foot protein 11; Tyr: Tyrosinase; BPP4: Byssal peroxidase-like protein 4; PPO: Polyphenol oxidase. Control: The solvent only control (0.5% DMSO in FSW). PA+PGPC: the mixture of 0.26 µg mL^-1^ PA and 0.96 µg mL^-1^ PGPC. Data shown are means ± standard error of replicates (n = 3).


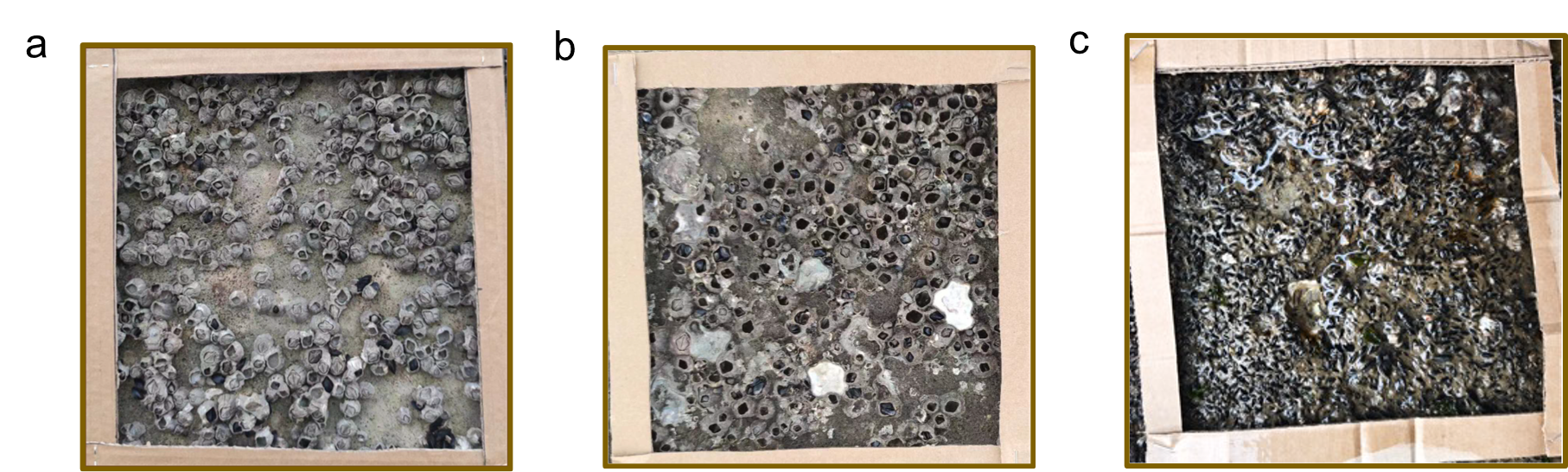


Figure S5. Examples of sampling zones used during the field surveys. a) A zone with high density of *B. albicostatus*. b) A zone with medium density of *B. albicostatus*. c) The zone with low density of *B. albicostatus*. The frame size is 20 cm x 20 cm.

**Table S1. Summary of abbreviations used in this study**

| Abbreviation | Description |
| --- | --- |
| FSW | Filtered (0.22 µm) seawater |
| DMSO | Dimethyl sulfoxide |
| BCS | *B. albicostatus* conditioned seawater |
| MCF | Mantle cavity fluid of *B. albicostatus* |
| PA | Palmitic acid |
| PGPC | 1-Palmitoyl-sn-glycero-3-phosphocholine |
| MGPC | 1-Myristoyl-sn-glycero-3-phosphocholine |
| NET | Norethindrone |
| LC-MS/MS | Liquid Chromatography–Tandem Mass Spectrometry |
| LC-MS | Liquid Chromatography-Mass Spectrometry |
| EIC | Extracted ion chromatogram |
| MFPs | Mussels secret foot proteins |
| SEM | Scanning Electron Microscopy |
| DOPA | 3,4-dihydroxyphenylalanine |
| SRA | Short Read Archive |
| DEGs | Differentially Expressed Genes |
| KEGG | Kyoto Encyclopedia of Genes and Genomes |
| PPO | Polyphenol oxidase |
| AFM | Atomic Force Microscopy |
| FPKM | Fragments Per Kilobase per Million reads |
| qRT-PCR | Quantitative real-time PCR |
| ANOVA | One-way analysis of variance |

**Table S2. Correlation coefficient between density of *B. albicostatus* and *V. atrata* in the three survey locations.**

| Survey location | Pearson’s correlation coefficient |
| --- | --- |
| Wuyuan Bay | -0.9981737 |
| Baicheng Bay | -0.9567846 |
| Haicang Bay | -0.9516158 |

Note: The Pearson’s correlation coefficient was calculated using the average density of *B. albicostatus* and *V. atrata* in each survey location by Excel 2018.

**Table S3.** **The 181 compounds detected in the MCF of *B. albicostatus* by LC-MS/MS**

|  | Name | Formula | m/z | Rt (s) | Relative abundance |
| --- | --- | --- | --- | --- | --- |
|  | 2-Oxoadipic acid | C_6_H_8_O_5_ | 141.01656 | 341.131 | 108559295 |
|  | Palmitic acid | C_16_H_32_O_2_ | 255.23343 | 38.857 | 9048248.24 |
|  | Choline | C_5_H_14_NO | 104.10644 | 222.397 | 8326153.93 |
|  | 1-O-Octadecyl-sn-glyceryl-3-phosphorylcholine | C_26_H_56_NO_6_P | 510.39096 | 195.943 | 4673097.45 |
|  | Erucamide | C_22_H_43_NO | 338.34126 | 35.3915 | 4160823.71 |
|  | Arg-Tyr | C_15_H_23_N_5_O_4_ | 337.1707 | 518.133 | 4115903.39 |
|  | Anthranilic acid (Vitamin L1) | C_7_H_7_NO_2_ | 138.05458 | 292.374 | 2738874.97 |
|  | Oleic acid | C_18_H_34_O_2_ | 281.24902 | 41.236 | 2398881.46 |
|  | N6,N6,N6-Trimethyl-L-lysine | C_9_H_20_N_2_O_2_ | 189.15916 | 536.378 | 1827285.88 |
|  | Sphingomyelin (d18:1/18:0) | C_41_H_83_N_2_O_6_P | 731.60507 | 178.5335 | 1593938.58 |
|  | L-Carnitine | C_7_H_15_NO_3_ | 162.11158 | 364.606 | 1473501.65 |
|  | Acamprosate | C_5_H_11_NO_4_S | 180.03347 | 148.7595 | 1252109.82 |
|  | EDTA | C_10_H_16_N_2_O_8_ | 293.09766 | 437.87 | 1218430.09 |
|  | Adenosine | C_10_H_13_N_5_O_4_ | 268.10378 | 170.765 | 1095567.35 |
|  | cis-9-Palmitoleic acid | C_16_H_30_O_2_ | 253.21755 | 53.8655 | 984755.633 |
|  | Thioetheramide-PC | C_45_H_93_N_2_O_4_PS | 758.56752 | 146.935 | 894444.109 |
|  | Heptadecanoic acid | C_17_H_34_O_2_ | 269.2485 | 52.146 | 731822.768 |
|  | 3-Methylhistidine | C_7_H_11_N_3_O_2_ | 170.09149 | 402.1295 | 696950.465 |
|  | 1-Palmitoylglycerol | C_19_H_38_O_4_ | 313.27294 | 35.308 | 637781.471 |
|  | Dimethylformamide | C_3_H_7_NO | 74.05945 | 21.229 | 558746.962 |
|  | Pristanic acid | C_19_H_38_O_2_ | 297.27973 | 40.517 | 557130.994 |
|  | Glycyl-L-leucine | C_8_H_16_N_2_O_3_ | 227.07891 | 320.979 | 547583.88 |
|  | L-Pyroglutamic acid | C_5_H_7_NO_3_ | 147.07522 | 398.852 | 530241.864 |
|  | Betaine | C_5_H_11_NO_2_ | 118.08527 | 398.933 | 442344.545 |
|  | D-Ornithine | C_5_H_12_N_2_O_2_ | 155.08087 | 261.6645 | 438099.042 |
|  | 1,2-dioleoyl-sn-glycero-3-phosphatidylcholine | C_44_H_84_NO_8_P | 786.59651 | 40.168 | 400304.985 |
|  | Norethindrone | C_20_H_26_O_2_ | 359.21955 | 72.5145 | 394829.278 |
|  | Citrate | C_6_H_5_O_7_^-3^ | 191.01937 | 491.2925 | 377564.664 |
|  | Myristic acid | C_14_H_28_O_2_ | 227.20155 | 39.52 | 374774.338 |
|  | Taurine | C_2_H_7_NO_3_S | 124.00671 | 299.428 | 373697.751 |
|  | 2-Methylbutyroylcarnitine | C_12_H_23_NO_4_ | 246.16908 | 242.15 | 351951.208 |
|  | Indoxyl sulfate | C_8_H_7_NO_4_S | 229.02917 | 79.444 | 318345.521 |
|  | 15-Deoxy-delta-12,14-PGJ2 | C_20_H_28_O_3_ | 317.21002 | 154.75 | 313623.769 |
|  | L-Arginine | C_6_H_14_N_4_O_2_ | 175.11818 | 400.254 | 278040.863 |
|  | 3-Methylphenylacetic acid | C_9_H_10_O_2_ | 149.06046 | 102.944 | 277865.576 |
|  | Lumichrome | C_12_H_10_N_4_O_2_ | 243.08702 | 64.235 | 266876.742 |
|  | Betaine aldehyde | C_5_H_12_NO+ | 162.11159 | 303.424 | 265036.34 |
|  | Diacetyl | C_4_H_6_O_2_ | 87.04326 | 6.901 | 234149.568 |
|  | Eicosapentaenoic acid | C_20_H_30_O_2_ | 325.21514 | 137.797 | 183938.86 |
|  | 1-Stearoyl-2-oleoyl-sn-glycerol 3-phosphocholine (SOPC) | C_44_H_86_NO_8_P |  | 832.58242 | 178899.838 |
|  | S-Methyl-5'-thioadenosine | C_11_H_15_N_5_O_3_S | 298.0957 | 100.2875 | 177391.552 |
|  | Glycerol 3-phosphate | C_3_H_9_O_6_P | 171.00604 | 386.749 | 155277.852 |
|  | Linoleic acid | C_18_H_32_O_2_ | 279.23315 | 40.517 | 145668.551 |
|  | (+-)5,6-DHET | C_20_H_34_O_4_ | 361.23599 | 137.47 | 144303.707 |
|  | 1-Myristoyl-sn-glycero-3-phosphocholine | C_22_H_46_NO_7_P | 468.30442 | 199.328 | 143532.407 |
|  | (+-)8,9-DHET | C_20_H_34_O_4_ | 361.23562 | 106.006 | 141712.255 |
|  | Cytosine | C_4_H_5_N_3_O | 112.04953 | 200.984 | 138906.656 |
|  | Thymine | C_5_H_6_N_2_O_2_ | 127.04918 | 101.151 | 134203.509 |
|  | N-Acetyl-L-glutamate | C_7_H_11_NO_5_ | 188.05585 | 394.7195 | 131853.817 |
|  | 1-Stearoyl-rac-glycerol | C_21_H_42_O_4_ | 359.31403 | 36.346 | 125726.628 |
|  | 1-Palmitoyl-sn-glycero-3-phosphocholine | C_24_H_50_NO_7_P | 496.33853 | 193.254 | 115150.903 |
|  | 1-Methylpseudouridine | C_10_H_14_N_2_O_6_ | 259.09148 | 173.354 | 113838.358 |
|  | Stearidonic Acid | C_18_H_28_O_2_ | 277.21471 | 58.324 | 113795.44 |
|  | Cholesteryl sulfate | C_27_H_46_O_4_S | 931.61973 | 28.854 | 111244.16 |
|  | 1-Oleoyl-sn-glycero-3-phosphocholine | C_26_H_52_NO_7_P | 522.3549 | 193.254 | 109898.472 |
|  | N2-Acetyl-L-ornithine | C_7_H_14_N_2_O_3_ | 175.11063 | 105.801 | 105603.104 |
|  | n-Propyl cinnamate | C_12_H_14_O_2_ | 208.13247 | 196.954 | 103775.007 |
|  | Glycerophosphocholine | C_8_H_20_NO_6_P | 258.1092 | 439.896 | 97822.1131 |
|  | 6-Hydroxydopamine | C_8_H_11_NO_3_ | 170.07783 | 364.199 | 97322.9248 |
|  | 4-Oxoretinol | C_20_H_28_O_2_ | 301.21483 | 152.809 | 97055.8266 |
|  | DL-3-Hydroxybutyric acid | C_4_H_8_O_3_ | 146.08012 | 407.456 | 96024.9153 |
|  | L-Histidine | C_6_H_9_N_3_O_2_ | 156.07565 | 444.6045 | 95562.863 |
|  | Ribothymidine | C_10_H_14_N_2_O_6_ | 257.07793 | 87.191 | 92624.5165 |
|  | (4Z,7Z,10Z,13Z,16Z,19Z)-4,7,10,13,1 6,19-Docosahexaenoic acid | C_22_H_32_O_2_ |  | 329.24611 | 92246.8501 |
|  | 1-Stearoyl-2-hydroxy-sn-glycero-3-phosphocholine | C_26_H_54_NO_7_P |  | 524.36902 | 90973.7946 |
|  | Acetylcarnitine | C_9_H_17_NO_4_ | 204.12232 | 304.946 | 86745.6165 |
|  | Erucic acid | C_22_H_42_O_2_ | 337.31068 | 41.176 | 86467.8881 |
|  | Glycylproline | C_5_H_10_N_2_O_4_ | 233.11266 | 432.9785 | 85882.0263 |
|  | 3-Phosphoserine | C_3_H_8_NO_6_P | 201.02589 | 44.076 | 82383.2236 |
|  | 5-Aminopentanoic acid | C_5_H_11_NO_2_ | 100.07464 | 400.3145 | 80886.285 |
|  | Creatinine | C_4_H_7_N_3_O | 114.06523 | 170.765 | 78979.866 |
|  | PGD2 | C_20_H_32_O_5_ | 335.22027 | 38.275 | 72943.0118 |
|  | PC(16:0/16:0) | C_40_H_80_NO_8_P | 734.56571 | 60.374 | 69196.5384 |
|  | L-Citrulline | C_6_H_13_N_3_O_3_ | 158.09114 | 463.74 | 68413.4502 |
|  | N.alpha.-Acetyl-L-lysine | C_8_H_16_N_2_O_3_ | 189.12223 | 372.552 | 68345.7889 |
|  | .beta.-Cyano-L-alanine | C_4_H_6_N_2_O_2_ | 97.03869 | 56.6955 | 66837.6207 |
|  | 16-Hydroxypalmitic acid | C_16_H_32_O_3_ | 295.22518 | 54.317 | 63712.7687 |
|  | Jasmine lactone | C_10_H_16_O_2_ | 151.11052 | 108.444 | 61828.8745 |
|  | Nicotinamide | C_6_H_6_N_2_O | 123.05401 | 64.715 | 61633.9595 |
|  | Quinolinate | C_7_H_5_NO_4_ | 231.04233 | 96.361 | 59929.8794 |
|  | 20-Hydroxyarachidonic acid | C_20_H_32_O_3_ | 303.23027 | 104.806 | 58842.0114 |
|  | 3-Deoxy-2-keto-6-phosphogluconic acid |  | 279.09019 | 36.666 | 55923.4356 |
|  | D-Mannose | C_6_H_12_O_6_ | 244.07839 | 257.405 | 53585.0815 |
|  | Caffeine | C_8_H_10_N_4_O_2_ | 236.11194 | 164.48 | 53448.9433 |
|  | Behenic acid | C_22_H_44_O_2_ | 339.3261 | 51.0655 | 52913.9039 |
|  | (3-Carboxypropyl)trimethylammonium cation |  | 146.11644 | 441.564 | 52313.6165 |
|  | D-Quinovose | C_6_H_12_O_5_ | 223.0817 | 310.1595 | 51914.6042 |
|  | Riboflavin | C_17_H_20_N_4_O_6_ | 377.14447 | 216.6305 | 51505.7302 |
|  | Thymidine | C_10_H_14_N_2_O_5_ | 243.09661 | 100.9725 | 51064.762 |
|  | Oxyquinoline | C_9_H_7_NO | 146.05903 | 68.4095 | 48319.73 |
|  | sn-Glycerol 3-phosphoethanolamine |  | 216.06223 | 395.5145 | 47772.8124 |
|  | Isopentenyladenosine | C_15_H_21_N_5_O_4_ | 336.16533 | 70.503 | 46531.029 |
|  | Deoxyinosine | C_10_H_12_N_4_O_4_ | 251.07816 | 116.5805 | 45323.9235 |
|  | Pantothenate | C_9_H_16_NO_5_ | 220.11669 | 278.662 | 44561.3269 |
|  | 2'-Deoxyuridine | C_9_H_12_N_2_O_5_ | 227.06707 | 117.188 | 44095.5294 |
|  | D-Fructose | C_6_H_12_O_6_ | 179.0557 | 393.737 | 40947.4097 |
|  | N-Docosanoyl-4-sphingenyl-1-O-phosphorylcholine |  |  | 787.66336 | 40200.3292 |
|  | Dihydrotachysterol | C_28_H_46_O | 381.34986 | 28.693 | 37670.6485 |
|  | L-homocysteic acid | C_4_H_9_NO_5_S | 182.01224 | 252.4465 | 37107.3294 |
|  | L-Glutamate | C_5_H_9_NO_4_ | 130.04889 | 403.9345 | 35039.7567 |
|  | Glycodeoxycholic acid | C_26_H_45_NO_6_ | 448.31024 | 54.024 | 34290.2392 |
|  | Histamine | C_5_H_9_N_3_ | 112.08552 | 371.367 | 34160.8707 |
|  | Pro-Val | C_10_H_18_N_2_O_3_ | 215.13773 | 371.663 | 32571.2114 |
|  | Dimethylglycine | C_4_H_9_NO_2_ | 104.06933 | 297.3305 | 31977.1522 |
|  | D-Erythrose 4-phosphate | C_4_H_9_O_7_P | 183.00406 | 305.832 | 30471.4446 |
|  | Arg-Ala | C_9_H_19_N_5_O_3_ | 246.15438 | 420.657 | 28884.7892 |
|  | 1-Indanone | C_9_H_8_O | 193.08508 | 106.19 | 27512.7085 |
|  | Phosphorylcholine | C_5_H_15_NO_4_P | 184.07235 | 387.175 | 27210.497 |
|  | Adenosine 3'-monophosphate | C_10_H_14_N_5_O_7_P | 348.06899 | 438.395 | 27162.4602 |
|  | Triethanolamine | C_6_H_15_NO_3_ | 210.13216 | 371.166 | 26654.7921 |
|  | L-Glutamine | C_5_H_10_N_2_O_3_ | 147.0753 | 439.6285 | 24888.591 |
|  | N-Acetylmannosamine | C_8_H_15_NO_6_ | 186.0752 | 105.0725 | 23398.6829 |
|  | Adenine | C_5_H_5_N_5_ | 136.06027 | 100.5875 | 23246.4784 |
|  | 3-methylcytidine | C_10_H_15_N_3_O_5_ | 258.10822 | 173.719 | 21972.9511 |
|  | Stearic acid | C_18_H_36_O_2_ | 567.53587 | 51.406 | 21034.08 |
|  | Deoxyadenosine | C_10_H_13_N_5_O_3_ | 252.10801 | 142.017 | 20413.8907 |
|  | 1-methylguanosine | C_11_H_15_N_5_O_5_ | 298.11309 | 199.342 | 19760.6706 |
|  | Lathosterol | C_27_H_46_O | 369.35039 | 162.0185 | 18882.3726 |
|  | 4-Guanidinobutyric acid | C_5_H_11_N_3_O_2_ | 146.09102 | 367.958 | 18842.8222 |
|  | Pentadecanoic Acid | C_15_H_30_O_2_ | 241.21702 | 21.547 | 18735.2025 |
|  | Leu-Leu | C_12_H_24_N_2_O_3_ | 245.18534 | 207.273 | 17386.3597 |
|  | L-Proline | C_5_H_9_NO_2_ | 116.06962 | 317.351 | 16748.5825 |
|  | 2'-O-methylguanosine | C_11_H_15_N_5_O_5_ | 298.11393 | 230.856 | 16335.2761 |
|  | Uracil | C_4_H_4_N_2_O_2_ | 113.0336 | 164.845 | 15724.6008 |
|  | Cholesterol 3-sulfate | C_27_H_46_O_4_S | 465.30429 | 79.799 | 15520.4921 |
|  | Xanthine | C_5_H_4_N_4_O_2_ | 151.0256 | 240.923 | 14412.697 |
|  | 1-O-(cis-9-Octadecenyl)-2-O-acetyl-sn-glycero-3-phosphocholine | C_28_H_56_NO_7_P | 550.38318 | 178.409 | 13557.7692 |
|  | Cyclohexylamine | C_6_H_13_N | 160.13141 | 316.441 | 13512.768 |
|  | N-Acetyl-D-lactosamine | C_14_H_25_NO_11_ | 384.14795 | 432.1085 | 13495.7619 |
|  | Hypoxanthine | C_5_H_4_N_4_O | 137.04486 | 218.6665 | 12934.3822 |
|  | Ile-Tyr | C_15_H_22_N_2_O_4_ | 312.19297 | 263.636 | 12213.7757 |
|  | D-Proline | C_5_H_9_NO_2_ | 116.06948 | 427.416 | 11472.5453 |
|  | Diethanolamine | C_4_H_11_NO_2_ | 70.06459 | 317.897 | 11427.2577 |
|  | Methylmalonic acid | C_4_H_6_O_4_ | 117.02218 | 372.149 | 11210.7232 |
|  | 6-Benzylaminopurine | C_12_H_12_ClN_5_ | 226.11379 | 109.68 | 10838.4065 |
|  | N-Acetylglucosamine 1-phosphate | C_8_H_16_NO_9_P | 300.04897 | 453.206 | 10774.5858 |
|  | L-Palmitoylcarnitine | C_23_H_45_NO_4_ | 400.34085 | 173.138 | 10451.9316 |
|  | N6-methyladenosine | C_11_H_15_N_5_O_4_ | 282.11856 | 132.8255 | 9145.39213 |
|  | .beta.-Homoproline | C_6_H_11_NO_2_ | 130.08521 | 387.795 | 8978.66483 |
|  | Arachidic acid | C_20_H_40_O_2_ | 311.29515 | 107.645 | 8928.008 |
|  | 2-Oleoyl-1-palmitoyl-sn-glycero-3-phosphocholine(PC(16:0/18:1(9Z))) | C_42_H_82_NO_8_P | 758.57207 | 41.77 | 8592.75957 |
|  | trans-Vaccenic acid | C_18_H_34_O_2_ | 283.26216 | 102.3985 | 8535.51635 |
|  | N2,N2-Dimethylguanosine | C_12_H_17_N_5_O_5_ | 312.12921 | 193.254 | 8014.40908 |
|  | Uridine | C_9_H_12_N_2_O_6_ | 245.07605 | 164.0675 | 7782.59906 |
|  | Indole-3-carboxylic acid | C_9_H_7_NO_2_ | 160.03995 | 91.208 | 7330.02003 |
|  | 3-Methyluridine | C_10_H_14_N_2_O_6_ | 259.09188 | 80.7715 | 7065.41087 |
|  | Guanosine | C_10_H_13_N_5_O_5_ | 284.09854 | 263.5655 | 6741.30451 |
|  | Kynurenic acid | C_10_H_7_NO_3_ | 188.03514 | 218.134 | 6455.47088 |
|  | Deoxyguanosine | C_10_H_13_N_5_O_4_ | 266.08982 | 171.1915 | 6324.73401 |
|  | 3'-O-methyladenosine | C_11_H_15_N_5_O_4_ | 282.11838 | 104.3925 | 6120.67532 |
|  | Arg-Arg | C_14_H_30_N_8_O_5_ | 394.23149 | 502.217 | 6107.02809 |
|  | Cytidine | C_9_H_13_N_3_O_5_ | 244.09217 | 242.15 | 5787.04276 |
|  | Cholecalciferol (Vitamin D3) | C_27_H_44_O | 367.33391 | 136.662 | 5684.76446 |
|  | 1-Palmitoyl-2-hydroxy-sn-glycero-3-phosphoethanolamine | C_21_H_44_NO_7_P | 454.28979 | 196.954 | 5433.93824 |
|  | Acadesine (Drug) | C_9_H_14_N_4_O_5_ | 317.11794 | 40.524 | 5407.98536 |
|  | Trimethylamine N-oxide | C_3_H_9_NO | 117.10192 | 272.6925 | 5357.73428 |
|  | Tyr-Met | C_14_H_20_N_2_O_4_S | 312.11028 | 170.765 | 4916.6959 |
|  | His-Glu | C_11_H_16_N_4_O_5_ | 285.11859 | 419.431 | 4647.40955 |
|  | N-Carbamylglutamate | C_6_H_10_N_2_O_5_ | 191.06531 | 417.721 | 4610.66287 |
|  | 2-Deoxyribose 5-phosphate | C_5_H_11_O_7_P | 250.96728 | 107.269 | 4371.17021 |
|  | Glutaraldehyde | C_5_H_8_O_2_ | 83.04829 | 406.722 | 4361.28273 |
|  | Desmosterol | C_27_H_44_O | 367.33403 | 106.095 | 4229.54879 |
|  | Allopurinol riboside | C_10_H_12_N_4_O_5_ | 269.08697 | 218.659 | 4187.70905 |
|  | Nicotinamide adenine dinucleotide (NAD) | C_21_H_27_N_7_O_14_P_2_ | 664.11401 | 439.44 | 3774.2905 |
|  | Tetracosanoic acid | C_24_H_48_O_2_ | 367.35711 | 111.029 | 3764.09602 |
|  | Sumatriptan | C_14_H_21_N_3_O_2_S | 372.06347 | 256.203 | 3706.34418 |
|  | D-Ribose 5-phosphate | C_5_H_11_O_8_P | 250.99802 | 29.665 | 3543.90294 |
|  | D-Lyxose | C_5_H_10_O_5_ | 187.0014 | 365.983 | 3166.07115 |
|  | Dihydroxyacetone phosphate | C_3_H_7_O_6_P | 229.01161 | 409.056 | 2372.4248 |
|  | alpha-D-Glucose 1-phosphate | C_6_H_13_O_9_P | 326.98858 | 361.938 | 2185.17709 |
|  | Gemcitabine | C_9_H_11_F_2_N_3_O_4_ | 228.0622 | 260.9325 | 1824.79613 |
|  | 2-Hydroxyadenine | C_5_H_5_N_5_O | 152.05531 | 231.561 | 1687.90296 |
|  | L-Phenylalanine | C_9_H_11_NO_2_ | 164.07134 | 271.3815 | 1209.38436 |
|  | Ile-Asn | C_10_H_19_N_3_O_4_ | 284.09793 | 288.2775 | 1095.7313 |
|  | Inosine | C_10_H_12_N_4_O_5_ | 269.08741 | 240.953 | 1077.83496 |
|  | Glycerol | C_3_H_8_O_3_ | 91.03949 | 107.142 | 1049.64951 |
|  | Deoxycytidine | C_9_H_13_N_3_O_4_ | 228.09657 | 208.799 | 767.581992 |
|  | .alpha.-L-Glu-L-Asp | C_9_H_14_N_2_O_7_ | 307.0438 | 234.3345 | 728.720968 |
|  | n-Octanamide | C_8_H_17_NO | 175.14841 | 56.538 | 680.758333 |
|  | Xanthurenic acid | C_10_H_7_NO_4_ | 428.11563 | 230.883 | 376.212717 |
|  | Oxypurinol | C_5_H_4_N_4_O_2_ | 133.01425 | 2.7 | 258.219232 |

**Table S4.** **The** **35 compounds tested for activity against *V. atrata* attachment, and the supplier and purity of their standard compounds**

| List | Compound | Supplier | Purity |
| --- | --- | --- | --- |
| 1 | D-Ornithine | Aladdin | ≥98% |
| 2 | Norethindrone | Aladdin | ≥98% |
| 3 | Citrate | Aladdin | ≥98% |
| 4 | Myristic acid | Aladdin | ≥95% |
| 5 | Lumichrome | Aladdin | ≥95% |
| 6 | S-Methyl-5'-thioadenosine | Aladdin | ≥95% |
| 7 | 1-Myristoyl-sn-glycero-3-phosphocholine | Aladdin | ≥99% |
| 8 | Cytosine | Aladdin | ≥98% |
| 9 | Thymine | Aladdin | ≥98% |
| 10 | N-Acetyl-L-glutamate | Aladdin | ≥99% |
| 11 | 1-Palmitoyl-sn-glycero-3-phosphocholine | Aladdin | ≥99% |
| 12 | 1-Methylpseudouridine | Aladdin | ≥98% |
| 13 | Betaine aldehyde | Aladdin | ≥95% |
| 14 | 2-Oxoadipic acid | ZZBIO | ≥98% |
| 15 | Oleic acid | ZZBIO | ≥99% |
| 16 | N6,N6,N6-Trimethyl-L-lysine | ZZBIO | ≥99% |
| 17 | Erucamide | ZZBIO | ≥99% |
| 18 | Palmitic acid | ZZBIO | ≥98% |
| 19 | L-Carnitine | ZZBIO | ≥98% |
| 20 | EDTA | ZZBIO | ≥98% |
| 21 | Adenosine | ZZBIO | ≥98% |
| 22 | Thioetheramide-PC | ZZBIO | ≥99% |
| 23 | Heptadecanoic acid | ZZBIO | ≥98% |
| 24 | 3-Methylhistidine | ZZBIO | ≥98% |
| 25 | 1-Palmitoyl glycerol | ZZBIO | ≥98% |
| 26 | Dimethylformamide | ZZBIO | ≥99% |
| 27 | Glycyl-L-leucine | ZZBIO | ≥99% |
| 28 | L-Pyroglutamic acid | ZZBIO | ≥95% |
| 29 | Betaine | ZZBIO | ≥95% |
| 30 | Choline | ZZBIO | ≥98% |
| 31 | L-Arginine | Sigma | ≥98% |
| 32 | Taurine | Sigma | ≥95% |
| 33 | Riboflavin | Sigma | ≥98% |
| 34 | Anthranilic acid (Vitamin L1) | Sigma | ≥95% |
| 35 | Uracil | Sigma | ≥99% |

**Table S5. Summary of RNA-seq data for *V. atrata* in the control (CON) group and the PA+PGPC group**

| Sample | Total raw reads | Total clean reads | Clean reads  Q20(%) | Clean reads Q30(%) | GC content(%) | |
| --- | --- | --- | --- | --- | --- | --- |
| CON 1 | 22520053 | 20914725 | 97.30 | 92.41 | 38.46 |  |
| CON 2 | 23241892 | 21350446 | 97.66 | 93.25 | 37.96 |  |
| CON 3 | 22165564 | 20317635 | 97.41 | 92.78 | 38.90 |  |
| PA+PGPC 1 | 24112226 | 22961939 | 97.46 | 92.77 | 38.45 |  |
| PA+PGPC 2 | 21516502 | 20063497 | 97.63 | 93.21 | 38.11 |  |
| PA+PGPC 3 | 22711458 | 21064659 | 97.74 | 93.48 | 39.29 |  |

**Table S6. Summary of the de novo assembly of the *V. atrata* transcriptome**

|  | Min Length (nt) | Mean length (nt) | Median length (bp) | Max length (bp) | N50 | Total number | Total length (nt) |
| --- | --- | --- | --- | --- | --- | --- | --- |
| Transcripts | 301 | 776 | 540 | 19268 | 915 | 435731 | 338224533 |
| Unigenes | 301 | 759 | 514 | 19268 | 898 | 175683 | 133387868 |

**Table S7. Results of the field survey on attachment of *B. albicostatus* on *V. atrata* and vice versa**

| Survey location | The proportion of *V. atrata* (n =300) with *B. albicostatus* attached to their shells | The proportion of *B. albicostatus* (n =300) with *V. atrata* attached to their shells |
| --- | --- | --- |
| Wuyuan Bay | 0.33 % | 0 % |
| Baicheng Bay | 0.67 % | 0 % |
| Haicang Bay | 0 % | 0 % |

Note: The three survey locations, Wuyuan, Baicheng, Haicang Bays are shown in Fig. 1a. In each survey location, 300 *V. atrata* and 300 *B. albicostatus* were randomly examined.

**Table S8. Details of primers used for qRT-PCR experiments**

| Gene ID | Gene | Primer Sequence (5’-3’) |
| --- | --- | --- |
|  | β-actin | F: AGATCTTGCAGGACGTG  ACC |
|  |  | R: GTGATTACTTGCCCGTC  AGG |
| Cluster-32477.56650 | Precollagen-NG | F: CACCTCCACCTCCAGCA  TTA |
|  |  | R: GGTGTGAACGGAAACCT  TGG |
| Cluster-32477.63190 | Mussel Foot Protein 2 | F: ACTGCCACAAAGACCGG  ATA |
|  |  | R: GGCATGGATTGGGATGA  CAC |
| Cluster-32477.53811 | Mussel Foot Protein 11 | F: GTGACGTTCAGCAAGTT  GGT |
|  |  | R: CGTGTCTAGGTTGAACA  GCG |
| Cluster-32477.28663 | Tyrosinase | F: GGCCTCCTCCTGAAGAT  GTT |
|  |  | R: GGCAAGCTTTCAGGACC  ATT |
| Cluster-32477.48912 | Byssal peroxidase-like protein 4 | F: GAGATACAAACGTGGTGA  CCG |
|  |  | R: AGGAGCGATGAACACTCT  TCT |
| Cluster-32477.119740 | Polyphenol oxidase | F: CGACTGTGAGGCCAAAC  AAA |
|  |  | R: AGTGTTGAGTCCCAGTA  CGG |
